# Superhydrophobic Wearable Strain Sensors: From Strategic Design to Robustness Paradigm

**DOI:** 10.1007/s40820-026-02220-w

**Published:** 2026-06-01

**Authors:** Haoyang Song, Yibo Liang, Guangying Zhang, Kaiqi Long, Ke Shi, Xinyu Han, Changsheng Liu, Yongquan Qing

**Affiliations:** 1 School of Materials Science and Engineering, Northeastern University, Shenyang, 110819 People’s Republic of China; 2 Key Laboratory for Anisotropy and Texture of Materials, Ministry of Education, Northeastern University, Shenyang, 110819 People’s Republic of China; 3 Foshan Graduate School of Innovation, Northeastern University, Foshan, 528312 People’s Republic of China; 4 Institute of Applied Ecology, Chinese Academy of Sciences, Shenyang, 110016 People’s Republic of China

**Keywords:** Wearable strain sensors, Superhydrophobicity, Robustness, Failure mechanisms, Amphibious sensing

## Abstract

Superhydrophobic wearable strain sensors represent an emerging frontier in flexible electronics, offering the potential to bridge the gap between laboratory prototypes and practical applications in humid, corrosive, or underwater environments. However, their widespread adoption is hindered by insufficient robustness against chemical, mechanical, and wetting state failures. The current literature lacks systematic insights into coupled multimode failures and integrated optimization frameworks, and standardized protocols for robustness evaluation remain absent. This review systematically summarizes strategies for material selection, structural design, and functional integration in superhydrophobic systems, with a focus on analyzing failure mechanisms across chemical, mechanical, and interfacial state dimensions. Key quantitative benchmarks—including resistance drift, contact angle retention, and cyclic stability—are established. We introduce a “failure-mechanism-oriented robustness optimization” framework and summarize corresponding testing standards. Finally, we discuss key future challenges and potential breakthroughs, most urgently the development of eco-friendly low-surface-energy modifiers and unified testing protocols, providing a theoretical framework and technological roadmap for the next generation of robust amphibious flexible sensing systems.

## Highlights

- The system analyzes coupled failure mechanisms from chemical, mechanical, and interfacial states, moving beyond the single‑failure‑mode focus of existing studies.
- A failure‑mechanism‑driven robustness optimization framework is established, defining key quantitative indicators to address the lack of unified optimization and evaluation criteria.
- Addressing sensor application bottlenecks, this review summarizes material–structural–functional integration strategies, identifies key future directions, and offers a practical theoretical framework and technical roadmap for next‑generation robust amphibious flexible sensing systems.

## Graphical abstract

**Figure:**
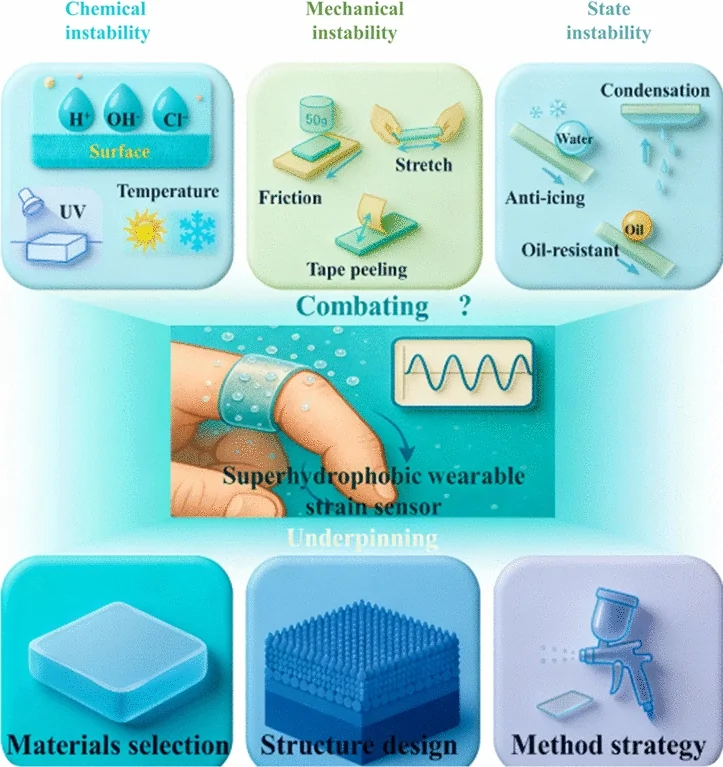

## Introduction

Flexible wearable strain sensors now serve as the primary conduit between physiological signals and digital systems, merging mechanical compliance, mass matching with the epidermis, and seamless wireless integration [1–4]. Among transduction schemes, resistive gauges outperform piezoelectric (responsive exclusively to dynamic stimuli), triboelectric (unstable and wear-prone), and capacitive (low sensitivity, EMI-vulnerable) alternatives in simplicity, cost, linearity, and wearing comfort [5–7]. Yet, as the physical gateway between skin and the outside world, these devices must operate in biochemical environments that are intrinsically hostile: Sweat ions (Na^+^, K^+^, Cl^−^) create parasitic conductance that drifts from the baseline [8]; chloride catalyzes oxidation and fracture of conductive networks, raising resistance by orders of magnitude [9]; and sebum, microbes, and airborne contaminants add interfacial impedances of 10^2^–10^3^ Ω [10]. Immersion, high humidity, freezing, or corrosive media accelerate these processes, producing transient or permanent failure. Conformal barriers suppress degradation, but always trade off gauge factor—the key merit of skin-like electronics [11, 12]. Hence, realizing wearable strain sensors that retain high gauge factors while remaining impervious to complex outdoor, clinical, and industrial conditions is no longer incremental—it is imperative.

Superhydrophobicity offers a paradigm shift in circumventing the long-standing environmental robustness bottleneck of stretchable electronics. By co-engineering low-surface-energy chemistries with micro-/nanoscale topography, a stable solid–air composite interface is created that acts as an ever-present, molecularly thin barrier against water and sweat [13–15]. Condensed droplets are repelled or roll off, spontaneously removing surface contaminants and preserving signal fidelity during splash, condensation, or transient immersion [16–20]. Unlike conventional bulk encapsulation, this surface-centric protection introduces no mechanical compliance penalty and, crucially, can be exploited for function: The same topographic features that generate super-repellency localize mechanical strain, thereby amplifying the piezoresistive response of embedded conductive networks [21–23]. The resulting architecture simultaneously delivers environmental tolerance, self-cleaning, and ultrahigh sensitivity without sacrificing stretchability, positioning it as an attractive substrate for outdoor textiles, disposable healthcare monitors, and epidermal human–machine interfaces that must survive harsh climate, repeated sterilization, or direct biofluid exposure [24–30].

**Fig. 1:**
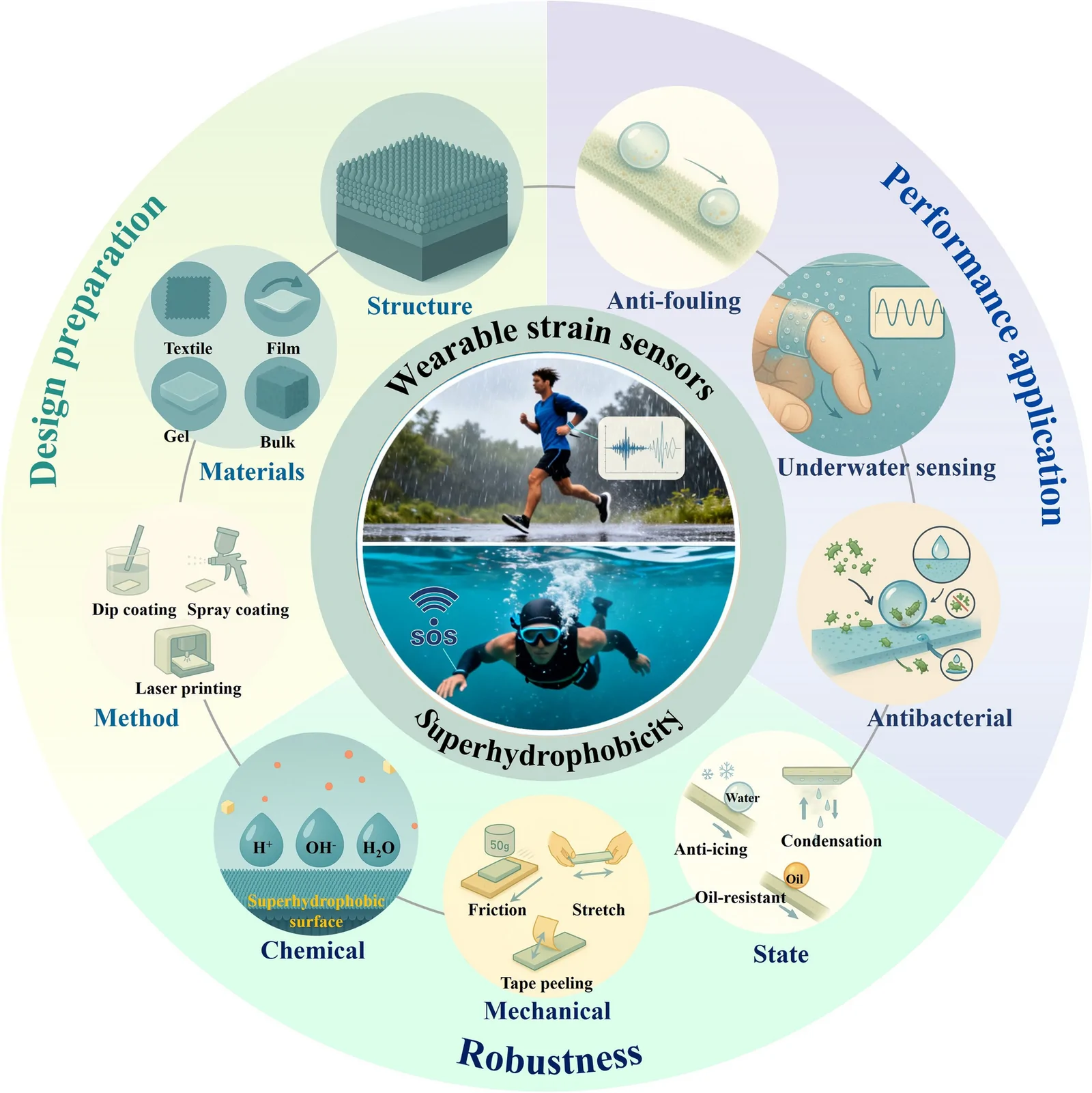
Schematic illustration of the design preparation, performance application, and robustness of a superhydrophobic wearable strain sensors

However, the long-term stability of superhydrophobic wearable strain sensors under harsh or end-use conditions has emerged as the critical bottleneck limiting their transition from laboratory demonstrations to field deployment (Fig. 1) [31–33]. Failure is seldom single mode; instead, it arises from three coupled pathways: (i) chemical decay. Prolonged exposure to acids, alkalis, oxidants, or salt fog hydrolyzes, oxidizes, or simply dissolves the low-surface-energy coating or the substrate beneath, producing a rapid drop in contact angle and loss of the air layer [34]; (ii) mechanical damage. Repeated stretching, twisting, rubbing, or impact fractures, buckles, or abrades the fragile surface roughness, reducing both water repellency and the local strain amplification that underpins sensor sensitivity [35]; the same damage sites then trap sebum or microbes that further accelerate aging [36]; and (iii) wetting state transition. External pressure, low-surface-tension liquids, frost-induced condensation or gradual surface energy increase can drive the irreversible Cassie-to-Wenzel transition, after which water fully wets the texture and the sensor no longer recovers its original baseline resistance [37, 38]. Therefore, a robust amphibious flexible strain sensor is defined as a wearable strain sensor capable of maintaining stable mechanical, electrical, and superhydrophobic properties in both air and underwater environments, as well as under prolonged exposure to sweat, high humidity, or bodily fluids. Shortfalls in chemical, mechanical, and wetting state robustness now dominate the commercialization roadmap of superhydrophobic wearable strain sensors. Piecemeal solutions—more robust coatings, tougher textures, pressure- or ice-resistant microstructures—have been reported [39–41].

At present, the systematic understanding of the coupling mechanisms between chemical, mechanical, and wetting failures—and their modulation by intrinsic material limitations, structural failure modes, and the kinetic/thermodynamic drivers of wetting transitions—remains fragmented and incompletely synthesized. Furthermore, the translation of these insights into a unified, synergistic stabilization strategy remains elusive. To bridge this critical gap, this review adopts a distinct, failure-centric analytical framework to systematically deconstruct the failure modes of superhydrophobic wearable strain sensors under chemical instability, mechanical degradation, and wetting state transition, alongside corresponding mitigation strategies. It begins by elaborating on the design principles governing the integration of high compliance, water repellency, and sensing sensitivity. Subsequently, by elucidating the quantitative benchmarks, synergistic interactions, and standardized evaluation criteria associated with these multidimensional failures, we establish a cohesive and robust failure-oriented paradigm. This framework provides a clear theoretical foundation and a practical technological roadmap, facilitating the development of flexible electronics capable of reliable operation in extreme and complex environmental conditions.

## Strategic Design of Superhydrophobic Wearable Strain Sensors

Superhydrophobic wearable strain sensors merge water-repellent surfaces with stretchable transduction [42]. By pairing material chemistry with microscale architecture, they retain high sensitivity, stretch and fast response even when exposed to moisture, sweat, or corrosive media, overcoming a key weakness of conventional flexible gauges [31]. This section begins with a survey of the elastomers, conductors, and fabrication routes underpinning these devices, followed by an outline of how surface roughness and low-energy coatings enable superhydrophobicity. Performance comparisons against nontextured analogues throughout highlight the enhancements in robustness and signal fidelity afforded by the superhydrophobic strategy.

### Material Elements: Substrates and Conductors

#### Flexible Substrate Materials

The flexible substrate serves as the supporting structure of a flexible wearable strain sensor and must maintain its integrity under various deformations such as bending, twisting, or stretching. The choice of substrate material directly dictates the material’s flexibility, stretchability, robustness, and the durability of its superhydrophobic properties—an essential advantage that overcomes the environmental limitations of conventional sensors [43]. An ideal substrate should combine high stretchability, chemical inertness, and surface modifiability. Current research focuses on elastomers and textiles, among other materials [44], with various flexible substrates being engineered to achieve elasticity, wearability, and robustness through specific treatments.

##### Flexible Elastomer Materials

Elastomers such as polydimethylsiloxane (PDMS) [45], Ecoflex [46], polystyrene-ethylene-butylene-styrene (SEBS) [47], natural rubber [48], polyurethane [1], and hydrogels [49] are commonly used as flexible substrates. They share key characteristics such as high stretchability, low modulus, good biocompatibility, and suitability for microstructural processing, making them naturally well suited for wearable strain sensors [50]. Among these, PDMS stands out as a preferred substrate for elastic strain sensors due to its optical transparency, chemical robustness, and low cost [51]. Its inherent low surface energy also facilitates subsequent hydrophobic modification, making PDMS suitable for use as a surface layer in superhydrophobic strain sensors. PDMS is prepared via emulsion blending [52] and templating methods [53], with laser-induced techniques [54] employed to construct surface structures, serving as a stable and high-performance elastic substrate.

**Fig. 2:**
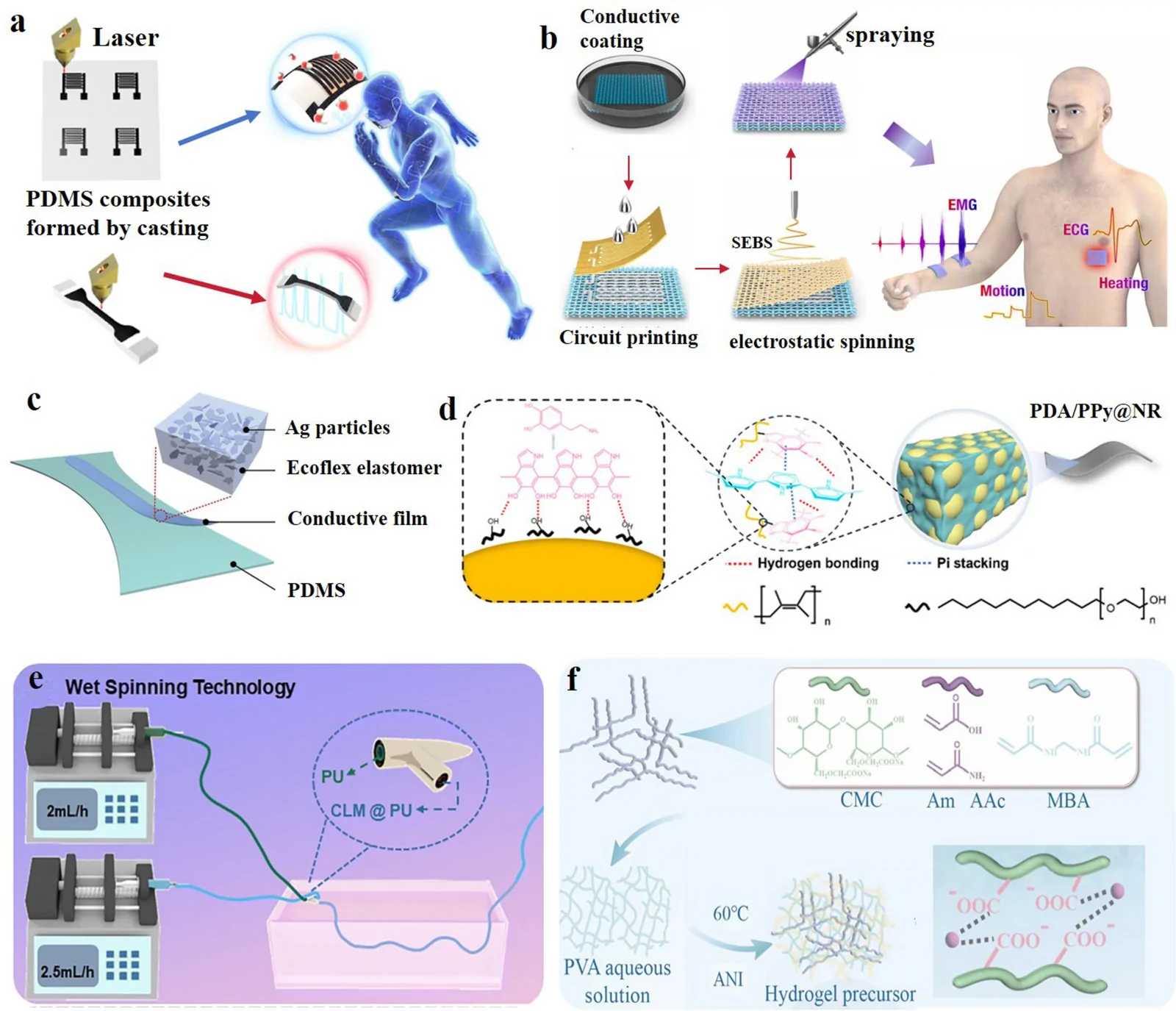
Types, characteristics, and common fabrication processes of flexible substrate materials. **a** Laser-printed graphene, polyether ether ketone powder, and PDMS-based elastic composite [55]. Copyright 2023, American Chemical Society. **b** Integrated health-regulating e-textile fabricated by electrospun-assisted layer-by-layer assembly and SEBS-based liquid metal printing [58]. Copyright 2023, Elsevier. **c** Ag–Ecoflex elastomeric conductive material printed using Ag filler–Ecoflex matrix ink on PDMS [65]. Copyright 2022, John Wiley and Sons. **d** Strain sensor based on a triple core–shell structure of polydopamine/polypyrrole@natural rubber [70]. Copyright 2024, John Wiley and Sons. **e** Strain sensor fabricated via coaxial spinning and spray coating with a polyurethane matrix [76]. Copyright 2024, Royal Soc Chemistry. **f** Hydrogel strain sensor composed of sodium carboxymethyl cellulose and acrylamide–acrylic acid–polyvinyl alcohol copolymer [91]. Copyright 2025, Springer Nature

The templating method employs physical structures as molds [53]. Functional materials are filled or deposited into the template voids, and after solidification, the template is removed to form micro-/nanostructures complementary to the mold. In contrast, laser-induced techniques enable direct and template-free patterning [54]. This approach typically uses a CO_2_ laser to irradiate polymers such as polyimide, where photothermal cleavage of chemical bonds and transformation of carbon hybridization states lead to the in situ formation of conductive architectures like laser-induced graphene (LIG). Emulsion blending involves uniformly mixing a flexible matrix precursor with functional components in emulsion form [52]. After coating and curing, a functional layer is formed on the flexible substrate. This process offers simplicity, low cost, scalability to large areas, and ensures uniform dispersion of functional fillers while maintaining compatibility with the flexible substrate. To achieve optimal performance, the fabrication of PDMS-based sensors often combines multiple processes. For example, one reported strain sensor was fabricated by laser-printing graphene on an elastic composite composed of polyether ether ketone powder and template-molded PDMS, followed by encapsulation **(**Fig. 2a) [55]. This sensor exhibited high sensitivity and stable resistance response across its working range, enabling detection of both subtle arterial pulses and larger strains. However, it relies on multimaterial heterogeneous interfaces and serial laser processing, resulting in fragile interface bonding, signal delay, and difficulty in balancing high sensitivity and consistency in large-scale production [56]. PDMS suffers from poor adhesion to many active layers; this is usually overcome by introducing SEBS interlayers or oxygen plasma priming [45].

SEBS exhibits excellent elasticity and ultraviolet (UV) resistance, along with good processability. It is commonly processed into matrix structures through solution blending [57], electrospinning [58], or templating methods [59]. Electrospinning uses a high-voltage field to draw a polymer solution or melt into a charged jet; as the solvent evaporates, nanofibers collect on a target to form a porous membrane with large surface area, good breathability, and high flexibility [58]. The membrane can serve directly as the substrate or be loaded with functional fillers to form a sensing layer that improves both sensitivity and mechanical endurance. Song et al. [60] laminated an ultrathin SEBS film to a SEBS–liquid metal composite layer, producing a sensor that operates from 0 to 680% strain. Electron tunneling across the thin SEBS combined with the percolated liquid metal network in the composite yields a bilayer that retains a high conductivity of 3.7 × 10^5^ S m^−1^ while remaining almost strain-insensitive (Fig. 2b). Nevertheless, SEBS lacks extreme environment resistance, requiring structural design and precise process control to ensure conductive network stability under high strain.

Compared to other materials, Ecoflex exhibits inherent antibacterial activity, enhancing the durability of sensors in biological environments. It also features a short curing time and adheres well to textiles or plastics, enabling rapid sensor fabrication. Common processing techniques include blend casting [61], templating [62], coating [63], and 3D printing [64], each with distinct advantages for fabricating strain sensors with different structures. Coating entails directly applying the Ecoflex mixture onto flexible substrates, followed by drying under controlled thickness and conditions to form a functional layer [63]. This approach is ideal for producing ultrathin, large-area sensing membranes, particularly for textile-integrated applications. 3D printing builds complex three-dimensional structures layer by layer using Ecoflex materials, often combined with functional inks [64]. This technique offers high flexibility and customizability for producing sensors with unconventional or multidimensional architectures. Yuan et al. [65] developed an Ag–Ecoflex–PDMS elastomer by printing Ag filler–Ecoflex matrix ink onto PDMS (Fig. 2c). The sensor exhibits exceptional dynamic response—minimal overshoot, high strain sensitivity, and low hysteresis—rendering it well-suited for human motion tracking, human–machine interfacing, and virtual reality applications. Its pronounced thermal expansion/contraction can, however, disrupt the percolative network, necessitating sub-micrometer-dimensional control during fabrication.

Natural rubber stands out among elastomeric materials due to its high resilience and low cost. It has been utilized in wearable strain sensors via techniques such as 3D printing [66], solution casting [67], coating [68], and vacuum filtration [69]. Vacuum filtration is a method that enables rapid and efficient solid–liquid separation by applying a vacuum to draw the filtrate through a filter membrane, leaving solid particles retained. Zhan et al. [70] fabricated a strain sensor based on a triple core–shell structure through in situ polymerization of polydopamine (PDA) and polypyrrole (PPy) coated on natural rubber nanospheres (Fig. 2d). The sensor exhibits notable sensitivity over a wide strain range (~ 800%) and long-term reliability (2500 cycles at 50% strain). It is capable of effectively detecting and capturing both subtle and large human motions. Natural rubber is susceptible to degradation in moist, high-temperature, or chemically aggressive environments, often requiring surface modification or the incorporation of antioxidants for enhanced durability [71].

Polyurethane is widely used in wearable strain sensors owing to its favorable abrasion resistance and chemical robustness. Common fabrication methods include solution casting [72], electrospinning [73], wet spinning [74], and dip coating [75]. In wet spinning, a polymer solution is extruded through a spinneret into a coagulation bath, where it solidifies into continuous fibers. This technique enables the production of fibers with specialized cross sections, including ultrafine or porous structures. Lin et al. [76] fabricated a triple-layer strain sensor via coaxial spinning and spray coating, using liquid metal/CNTs–polyurethane as the electrode layer and spray-deposited wrinkled MXene as the functional surface; the sensor detects pressure changes at 0–200% strain and holds potential for wearable health and environmental monitoring (Fig. 2e). Thermoplastic polyurethane (TPU), a variant of polyurethane, can be processed into wearable strain sensors with high stretchability, robustness, and fast response via electrospinning, 3D printing, or templating methods [77–80]. However, TPU tends to lose its elasticity and sensing performance at elevated temperatures, limiting its use in high-temperature environments. In terms of large-scale manufacturing, dip coating generally has the highest production efficiency due to its simplicity, scalability, and rapid processing capability, while also exhibiting relatively stable interbatch performance consistency with automated process control [81]. Electrospinning and laser processing have lower production efficiency; their interbatch consistency is affected by process parameters and equipment stability, respectively. However, unified quantitative data and standardized evaluation criteria for production efficiency and interbatch consistency of these processes are still lacking.

Hydrogels derived from natural biopolymers—including polysaccharides, proteins, and nucleic acids—exhibit high transparency, biocompatibility, degradability, and self-adhesion. These attributes position them as ideal substrates for biointegrated sensors [82]. In addition to common synthetic polymers such as polyvinyl alcohol (PVA) [83, 84] and polyacrylamide [85], biopolymers—including polysaccharides (e.g., cellulose [86, 87], silk fibroin [88], chitosan [89]) and proteins [90]—have been employed as eco-friendly, biodegradable, and biocompatible hydrogel materials for stretchable electronics. To achieve multifunctionality, hydrogel sensors often incorporate multiple matrix materials. Han et al. [91] fabricated a high-performance hydrogel strain sensor by interpenetrating sodium carboxymethyl cellulose into a gel matrix composed of acrylamide, acrylic acid, and PVA (Fig. 2f). Through simple coordination with polyaniline and zinc chloride, the resulting hydrogel demonstrated remarkable freezing resistance and moisture retention, alongside stable mechanical flexibility over a broad temperature range. This sensor can monitor body movements such as elbow, finger, wrist, and knee bending, and also exhibits responsiveness to temperature, sweat, and pH variations, highlighting its potential as a multifunctional sensing substrate. The inherent hydrophilicity of biobased hydrogels conflicts with achieving superhydrophobicity. A synergistic strategy—combining surface micro-/nanostructuring with low-surface-energy modification—offers a solution: Leveraging biopolymer self-assembly to build hierarchical roughness, followed by surface functionalization, enables internal hydrophilicity to coexist with surface superhydrophobicity [92]. However, challenges persist, including limited mechanical strength, poor coating adhesion, and unstable conductive networks in humid environments. Recent advances address these through double-network cross-linking to enhance robustness, intermediate adhesive layers to improve interfacial bonding, and covalently integrated conductive network–biopolymer systems to ensure wet stability.

Recent hydrogel sensor advances address toughness, cyclic stability, and adhesion for wearable reliability [93]. Syndiotactic structures enhance durability via energy dissipation, yielding high tensile strength and cyclic stability—overcoming conventional brittleness for sustained motion monitoring [94]. MXene-reinforced hydrogels improve adhesion via hydrogen bonding with MXene nanosheets, while enabling fast gelation and rapid electrical response to mechanical stimuli, ensuring stable skin contact and real-time detection of subtle physiological signals and complex motions [95].

##### Textile Substrate Materials

Textile substrates encompass both natural fibers (e.g., cotton, silk, wool) and synthetic fibers. Natural fibers are generally not suitable for direct use in sensors due to their limited elasticity. In contrast, synthetic fibers are widely employed owing to their affordability, design flexibility, mechanical strength, skin compatibility, and inherent flexibility [96]. Fabrics naturally possess excellent flexibility and breathability, conform comfortably to body contours, and are thus well- suited for long-term wearable monitoring [97].

**Fig. 3:**
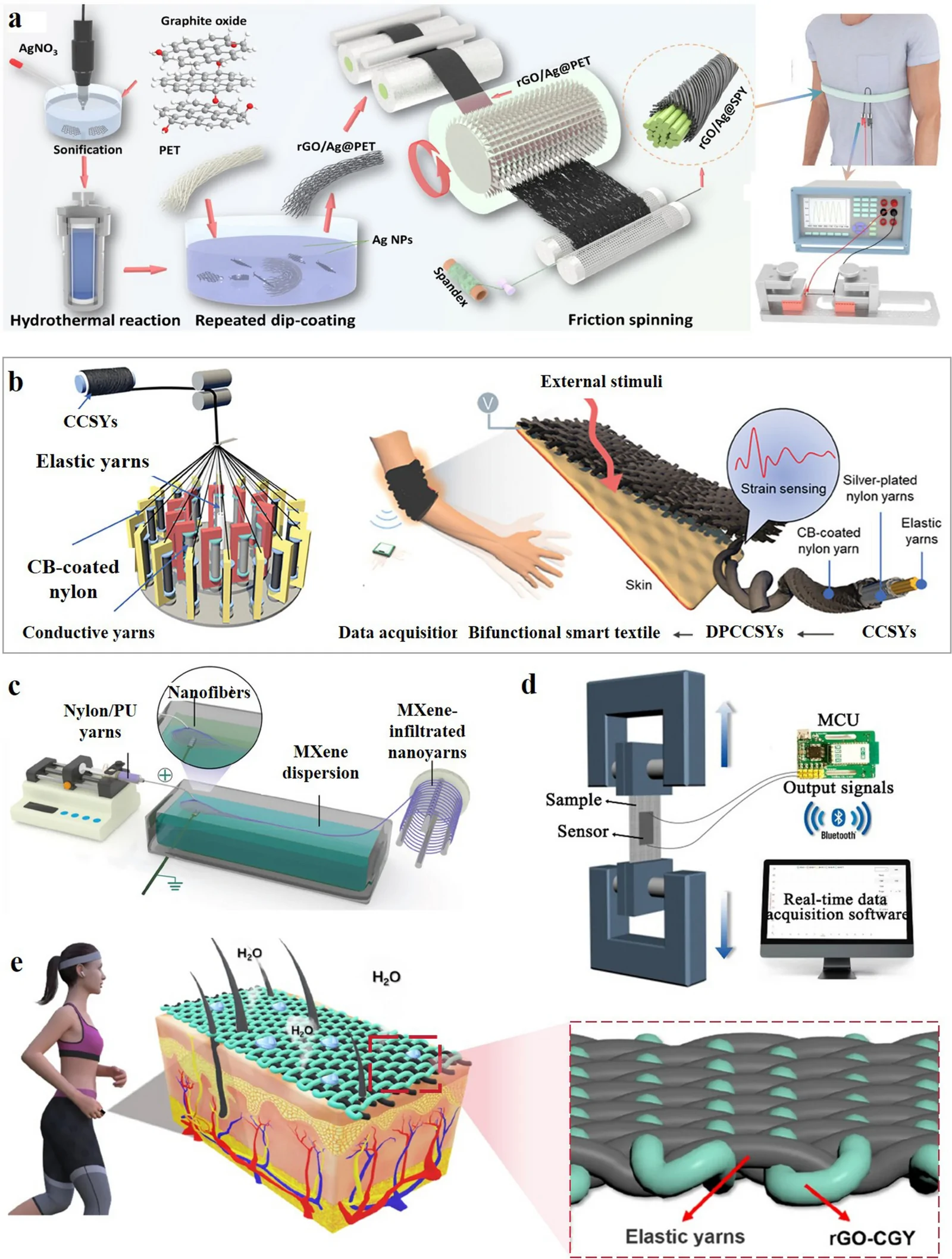
Textile substrates for wearable strain sensors: material choices, key properties and scalable processing routes. **a** Core–sheath yarn produced by friction spinning of rGO/Ag nanoparticles coated polyester around spandex filament [102]. Copyright 2024, Elsevier. **b** Sensing yarn comprising carbon black-coated nylon with silver-plated nylon and elastic spandex [104]. Copyright 2023, John Wiley and Sons. **c** MXene/polyurethane nanofibrous yarn formed by wet electrospinning nylon–polyurethane mats that capture MXene flakes [108]. Copyright 2020, John Wiley and Sons. **d** Photograph of a wearable strain sensor under tensile testing [107]. Copyright 2021, Elsevier. **e** Fabric-like strain sensor woven from graphene-modified Calotropis gigantea yarn interlaced with spandex in a free-standing cross-architecture [113]. Copyright 2023, Springer Nature

Polyester stands out in harsh environments due to its high corrosion resistance and mechanical robustness [98]. It also exhibits excellent thermal molding performance and dimensional robustness, resisting deformation under temperature variations or washing. The ester backbone of polyester confers robust oxidative and microbial stability, enabling durable, long-term wearable operation. Polyester-based strain sensors are commonly fabricated using methods such as coating [99], dip coating [100], lamination [101], and friction spinning [102]. The coating method involves applying a mixture of conductive materials, binders, and solvents onto polyester fibers, yarns, or fabrics by spraying, blade coating, or roller coating, followed by drying and curing to form a conductive composite. In contrast, dip coating utilizes the porous and wettable surface of polyester substrates. By immersing them into a dispersion of conductive materials, particles adhere uniformly to the fiber surface and internal pores via capillary action and adsorption, forming a stable conductive layer after drying. Lamination employs polydopamine as an adhesive to assemble layered structures of polyester/polyurethane fabric with conductive materials with different structures, consolidated by hot pressing [101]. Friction spinning, on the other hand, wraps rGO/Ag nanoparticles/polyester fibers around a spandex core via frictional forces, producing a core–sheath yarn (Fig. 3a) [102]. However, polyester fibers inherently lack elasticity and flexibility, limiting their adaptability to large deformations in wearable devices [98].

Nylon’s abundant amide linkages (–CONH–) impart high tensile strength and rapid elastic recovery, accommodating the complex kinematics of human motion, while its superior chemical inertness and wear resistance ensure reliable operation in harsh environments [103]. Liu et al. [104] developed a wearable fabric strain sensor by weaving core–sheath sensing yarns composed of carbon black-coated nylon (sheath) and silver-coated nylon combined with elastic spandex (core). The resulting sensor demonstrated high sensitivity, linearity, and durability, enabling effective monitoring of joint bending and potential injuries (Fig. 3b). Commonly used fabrication methods for nylon-based textile sensors include dip coating [105], blade coating [106], spun yarn weaving [107], and electrospinning [108]. The spun yarn weaving technique involves twisting short fibers into continuous yarns, which are then interlaced to form textiles. This approach allows efficient utilization of short fibers and offers structural diversity and enhanced mechanical properties to the fabric (Fig. 3c) [107]. Single-component nylon lacks sufficient extensibility for large-scale human strain and requires combination with high-elasticity fibers like spandex to meet wearable device demands [103].

Spandex-based wearable strain sensors are typically fabricated using techniques such as dip coating [109, 110], electrodeposition [111], and core-spun spinning [112]. The electrodeposition process utilizes an external electric field to drive the directional movement of charged particles, which undergo redox reactions on the electrode surface to form a deposited layer. This method offers the advantage of producing uniform, dense, and strongly adherent coatings on substrates with complex shapes, and it exhibits good real-time sensing data under tension (Fig. 3d). Zhang et al. [113] produced a textile-based strain sensor by cross-weaving spandex with Calotropis gigantea yarn, followed by dip coating with modified graphene (Fig. 3e). By positioning the sensor at different locations on the human body, they demonstrated its capability for full-range body-area monitoring of various physical movements and physiological signals, such as speaking, coughing, breathing, and walking. Spandex fabrics have limited functionality, and their high elasticity often damages the conductive layer, while fiber swelling during processing compromises modification effectiveness [114].

Elastomers and textiles share low modulus, high stretch, skin-like breathability, and proven biocompatibility, making them interchangeable soft substrates for wearable sensors [115]. However, each of these materials possesses distinct characteristics, necessitating the selection of appropriate processing techniques and structural configurations, along with the integration of suitable conductive components. Currently, research on advanced composite fibers is evolving from conventional piezoresistive responses toward multifield coupling and anisotropic sensing to meet the demands of detecting complex human motions.

The introduction of magnetorheological materials enables reversible modulation of stiffness and electrical conductivity under magnetic fields, facilitating programmable actuation and tunable sensitivity [116]. Moreover, composites with aligned conductive networks exhibit direction-dependent electrical responses, enabling accurate discrimination of complex deformations such as bending and twisting [117]. These mechanisms extend sensing capabilities from single-parameter detection to multifield response and directional recognition, offering new insights for the development of high-performance wearable devices. Building on this, textile substrates for wearable sensors are integrating multifunctional regulation with advanced spinning techniques to meet practical demands. The incorporation of phase change materials (PCMs) enables active thermal management by buffering temperature fluctuations. Encapsulating PCMs in flexible fibers allows reversible heat absorption and release, enhancing wearing comfort without sacrificing flexibility [118]. Advanced electrospinning, particularly conjugated electrospinning, enables core–shell or multicomponent fiber structures that optimize synergy between conductive materials and flexible matrices. For instance, constructing fiber networks anchored with conductive microspheres via conjugated electrospinning forms stable conductive pathways, improving sensitivity, response speed, and multidirectional strain recognition for precise motion detection [119].

#### Conductive Materials

A wearable strain sensor transduces mechanical deformation into a readable electrical signal; the conductor is therefore the critical element that sets sensitivity, response time, cycling life, and environmental robustness [120]. Current research focuses on three material families—metals, carbon allotropes, and conducting polymers—whose performance is optimized through nanostructuring, percolative networking or hybridization rather than by changing chemistry alone [97].

##### Metallic Nanomaterials

**Fig. 4:**
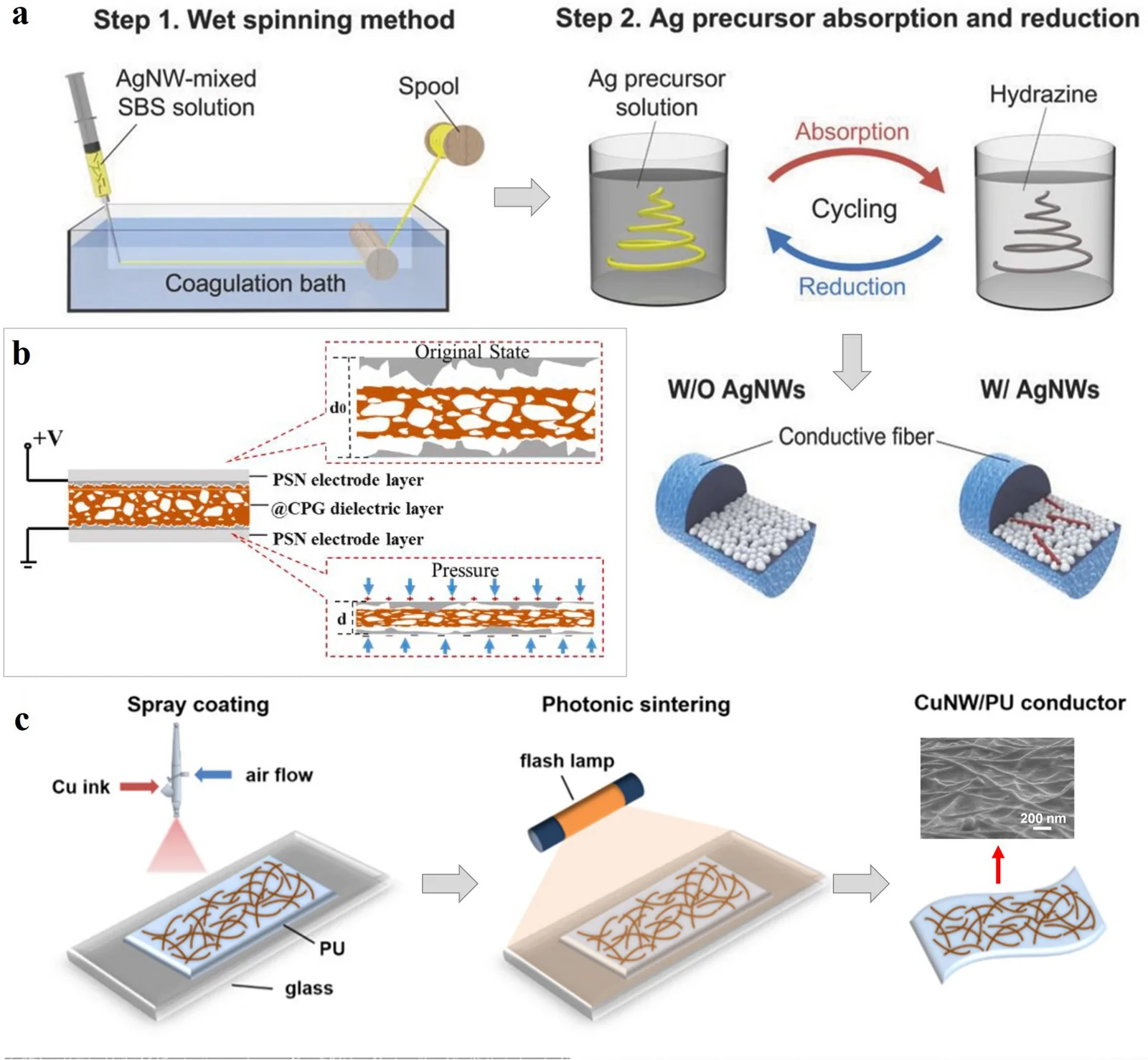
Metallic nanoconductors: variants, properties and scalable processing. **a** Highly stretchable fiber spun by wet spinning Ag nanowires and nanoparticles into an elastomer matrix [129]. Copyright 2015, John Wiley and Sons. **b** Flexible pressure sensor employing Ag–nanowire sandwich architecture that combines high gauge factor with 76% linear range [130]. Copyright 2024, Elsevier. **c** Cu–nanowire/polyurethane conductor fabricated by flash photonic sintering, yielding a 1.07 × 10^4^ S cm^−1^ track on a flexible substrate in milliseconds [131]. Copyright 2016, American Chemical Society

Metallic nanoconductors, such as Ag nanoparticles (AgNPs) [121], Ag nanowires (AgNWs) [122], copper nanoparticles (CuNPs) [123], and liquid metal [124], offer near-bulk conductivity and are therefore the default choice for high-sensitivity devices. AgNWs [125], for example, combine aspect ratios > 1000 with mechanical flexibility and optical transparency, and are routinely transferred to matrix material by spray, spin, or vacuum filtration [52, 126]; they are further stacked or hybridized to widen the strain window [127, 128]. Lee et al. [129] wet-spun AgNWs and AgNPs into a styrene–butadiene–styrene elastomer fiber that retains an initial conductivity of 2450 S cm⁻^1^ to 900% elongation; five such fibers integrated into a glove decode sign language in real time (Fig. 4a). The inherent ductility of the metal network delays crack initiation, giving sensors that survive thousands of stretch–release cycles without open-circuit failure. Owing to their unique physicochemical properties, metallic nanomaterials remain a pivotal material platform in the field of flexible wearable sensors. Architectural tuning, surface functionalization, and rapid low-temperature processing have already removed several limits: A AgNWs sandwich laminate delivers a gauge factor of 1254 across 0–76% strain (Fig. 4b) [130], and flash photonic sintering of Cu–nanowire inks yields 1.07 × 10^4^ S cm⁻^1^ tracks on heat-sensitive films in milliseconds (Fig. 4c) [131]. Nevertheless, metallic nanoconductors face key limitations in wearable strain sensors, including oxidation in humid environments, weak adhesion to flexible substrates, high fabrication costs, and poor dispersion. Liquid metals are prone to leakage under strain, requiring complex encapsulation. Their high density also increases sensor weight and stiffness, reducing comfort and flexibility [132].

##### Carbon-Based Conductive Materials

Compared with metal nanomaterials, carbon-based materials exhibit superior interfacial adhesion, oxidation resistance, and structural sustainability. These advantages make them particularly suitable for complex application scenarios [78]. Carbon-based conductive materials primarily encompassing 0D carbon black, 1D CNTs, 2D MXene, 2D graphene, and rGO, among others [133–137]. Within the carbon-based conductive material system, different structures confer unique performance advantages.

**Fig. 5:**
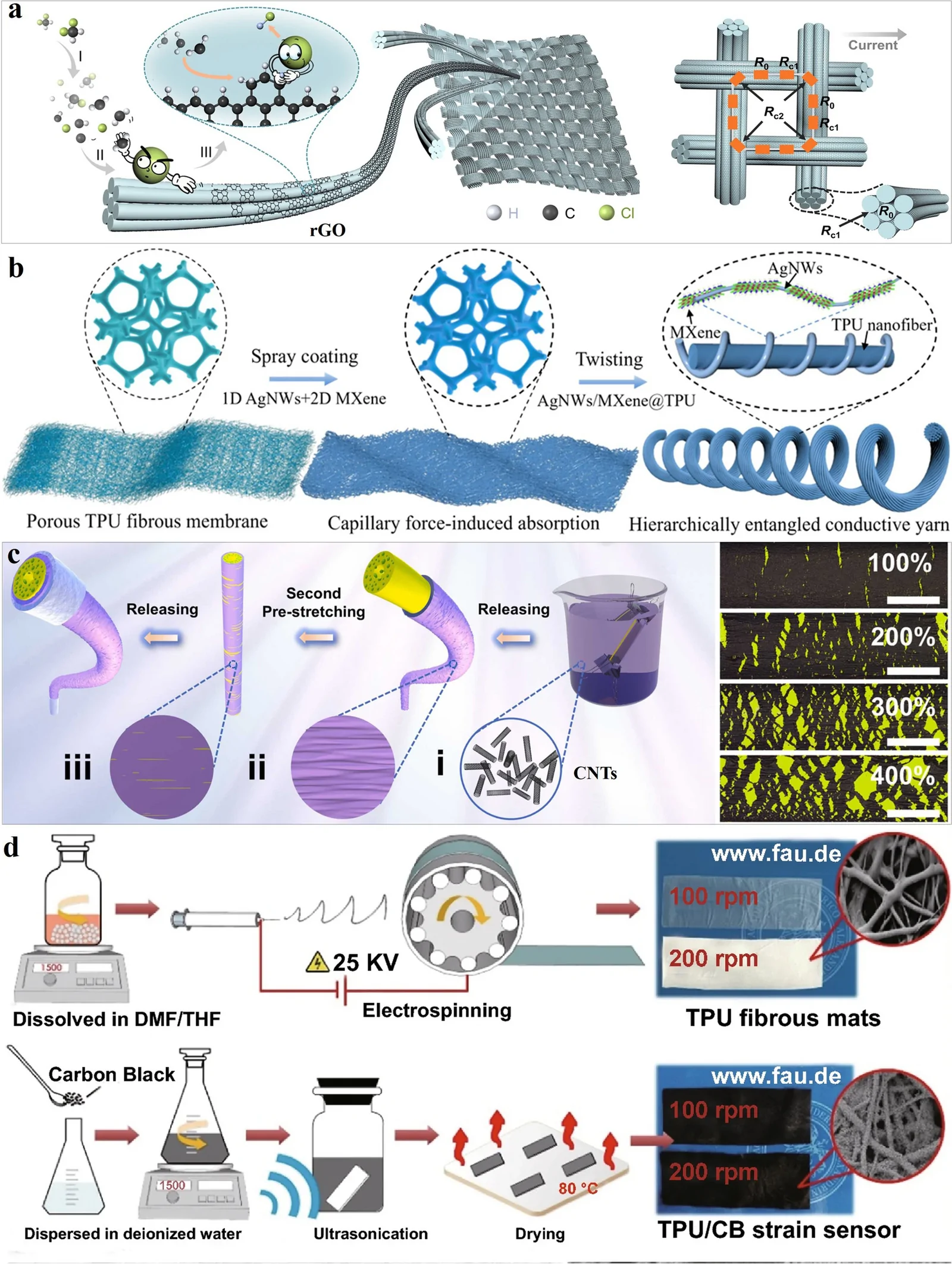
Carbon-based conductive nanomaterials: synthesis routes and architectures. **a** CVD-grown graphene on glass fiber fabric for ultrasensitive pressure sensing [147]. Copyright 2024, Springer Nature. **b** Hierarchical interlocked helical yarn spun from over-twisted AgNW/MXene sheaths around electrospun TPU nanofibers [162]. Copyright 2023, Elsevier. **c** Pre-strained, wrinkled CNTs layer on porous TPU fiber for fiber-type strain detection [190]. Copyright 2023, Elsevier. **d** Electrospun TPU mat embedded with carbon black particles yielding high-sensitivity strain sensors [78]. Copyright 2021, Springer Nature

Graphene’s two-dimensional, sheetlike architecture and its propensity to stack in well-defined multilayers endow the material with exceptional mechanical strength and flexibility while preserving highly stable, high-magnitude electrical conductivity [133, 138]. It is commonly fabricated via chemical vapor deposition (CVD) [139], redox methods [140], or liquid-phase exfoliation (LPE) [141], and integrated with flexible substrates through transfer printing, coating, or embedding techniques to form continuous conductive films or multilayer sandwich structures, thereby maintaining conductive pathway robustness under strain [142–146]. The essence of the CVD method involves the decomposition or chemical reaction of carbon-containing gaseous precursors on the surface of high-temperature metal catalyst substrates, generating carbon atoms that deposit onto the substrate surface and ultimately forming a graphene crystal structure through a cooling process. Wang et al. [147] grew graphene directly on glass fiber fabric (GGFF) by CVD, using dichloromethane as the carbon source (Fig. 5a). In situ generated Cl radicals promote CH_2_–Cl co-adsorption and accelerate H abstraction at graphene edges, yielding a continuous film at lower temperature. The resulting lightweight GGFF retains the weave’s hierarchical roughness and delivers pressure sensitivity sufficient to resolve pulse and vocal cord vibrations. However, CVD requires harsh high-temperature/vacuum conditions and noble metal catalysts, while transfer processes risk film damage.

LPE provides a scalable alternative: Ultrasound or shear disrupts the van der Waals bonding of graphite in low-surface-tension solvents, producing few-layer graphene dispersions without high temperature or metal catalysts [148]. Graphene can also be utilized in strain sensors via laser-induced processing. Yoon et al. [149] reported a full-range body strain sensor capable of detecting strains from ultrasmall to large magnitudes. The sensor was fabricated by laser engraving a graphene oxide–polyimide composite film to form rGO–LIG. This system effectively monitors various signals such as physiological pulses, speech waveforms, body movements, and American Sign Language recognition. The homogeneous rGO–LIG structure holds promise for enabling new functionalities in wearable electronics, bionic robots, soft robotics, and smart prosthetics. LPE also suffers from drawbacks such as easy re-aggregation, low exfoliation efficiency, and the production of low-quality multilayer graphene [150]. Moreover, graphene faces key challenges: Aggregation due to van der Waals forces disrupts conductivity and sensitivity, poor adhesion causes delamination under cyclic deformation, and high-quality large-scale production remains costly.

MXene—typically Ti_3_C_2_T_X_ transition metal carbides/nitrides—combines a 2D layered architecture with metallic conductivity (> 10^4^ S cm⁻^1^) and densely populated surface terminations (–O, –OH, –F). These attributes render it an ideal conductive phase for high-sensitivity, skin-mountable strain sensors [151]. The metallic band structure guarantees minimal sheet resistance, whereas the polar terminations impart water compatibility and facile chemical post-modification, enabling stable, high-solid-content inks that are compatible with low-temperature solution processing [152, 153]. It also benefits from the advantages of its structure and surface functional groups, making it easy to achieve superhydrophobic functionalization. MXene dispersions are often integrated with flexible substrates [154] via spin coating [155], spraying [156], vacuum filtration [157], screen printing [158], or dip coating [159]. To enhance sensing range and sensitivity, Wang et al. [160] designed a wearable strain sensor based on electrostatic adsorption and hydrogen bonding interactions, composed of TPU, MXene, PPy, and waterborne polyurethane. In this structure, MXene (2D) and PPy (3D) are uniformly distributed on the surface of MXene/TPU fibers, forming conductive and sensing pathways. This multidimensional composite strategy enables the fiber sensor to achieve both a wide sensing range and high sensitivity [161]. Drawing inspiration from climbing vines, Zhang et al. [162] over-twisted AgNW/MXene helical yarns around an elastomeric TPU–nanofiber scaffold (Fig. 5b). The high-aspect-ratio AgNW bridges MXene nanosheets into a percolated 3D network that interlocks with the stretchable TPU frame; the resulting trapezoidal helical architecture retains a conductivity of 1.1 × 10^5^ S m⁻^1^ at 300% strain. Beyond yarns, porous [163, 164], cracked [165–167], sandwich [168, 169], and wrinkled [170, 171] motifs are routinely introduced to MXene-based films to extend their sensing range. However, MXene faces critical challenges including susceptibility to oxidation upon prolonged air exposure, delamination of its layered structure under high strain, and the high cost of handling etching wastewater during large-scale production [172–174]. To address these issues, future research should focus on developing core–shell architectures with antioxidant coatings to shield MXene from oxygen, introducing dynamic cross-linkers to enhance interlayer interactions and structural stability, and optimizing green etching processes to reduce environmental impact.

CNTs leverage their one-dimensional tubular morphology, *sp*^2^-hybridized carbon atom network, and controllable alignment or random interwoven structures to significantly enhance the tunneling effect and interfacial deformation response in strain sensing [175]. This combination allows CNT-based functional layers to withstand repeated bending, stretching, and twisting on flexible substrates without brittle fracture, ensuring sensor durability and reliability [176, 177]. Moreover, CNTs possess high carrier mobility and electrical conductivity, providing sensors with low initial resistance and rapid electrical signal response—essential for detecting dynamic and subtle strains [178, 179]. In CNTs network-based sensors, electrical conduction occurs via tunneling between adjacent nanotubes [180]. CNTs, being inherently flexible nanomaterials, can be effectively integrated with various flexible substrates [181, 182] to form conformal contact. This compatibility helps avoid issues such as delamination or crack formation that often occur with rigid materials under bending. Common integration methods include spraying [183], dip coating [184], CVD [185], vacuum filtration [186], and 3D printing [187]. CNTs are particularly suitable for constructing microporous networks [188–190], crack-based motifs [191], wrinkled/wavy architectures [192, 193], and multilayer mesh structures [169, 194]. These configurations enable significant resistance variation even under minimal strain while maintaining high cyclic durability. Zhao et al. [190] employed a two-step pre-stretching strategy to incorporate both wrinkled and cracked structures in a CNTs conductive layer into a hollow porous TPU fiber (Fig. 5c). Benefiting from this synergistic conductive architecture, the resulting fiber strain sensor achieved a wide sensing range, high sensitivity, low detection limit, and satisfactory sensing robustness and durability. CNTs suffer from strong van der Waals forces, which induce aggregation and poor dispersion, disrupting conductive networks; weak substrate adhesion leads to delamination under cyclic deformation; high-purity production remains costly; chemical inertness limits modification; tunnel conduction causes signal instability under extreme conditions; and the tubular structure is prone to fracture, compromising long-term durability [180].

Carbon black, produced by incomplete combustion or pyrolysis of hydrocarbons, consists of near-spherical amorphous carbon nanoparticles that aggregate into branched chains or grape-like clusters [195, 196]. It serves as a conductive filler in strain sensors: Once the volume fraction exceeds the percolation threshold, the aggregates are separated by < 10 nm gaps, providing tunneling pathways throughout an insulating elastomer [197]. Because the particles readily agglomerate and microcrack, composites are prepared by pre-dispersing carbon black in a polymer precursor or solution [198, 199], followed by casting, electrospinning or freeze-drying and subsequent curing. Wang et al. [78] developed a highly sensitive strain sensor by embedding carbon black particles into an electrospun TPU fiber film (Fig. 5d). The resulting three-dimensional scaffold network enabled the sensor to achieve high gauge factor (GF of 8962.7 at 155% strain), fast response time, excellent robustness and durability, and a wide working range. Carbon black can significantly enhance the sensitivity, operating range, and linearity of strain sensors, making them suitable for diverse applications—from detecting subtle physiological signals to monitoring large-scale human motions [200, 201]. However, carbon black faces key limitations: Weak substrate adhesion causes slippage and signal drift; low conductivity forces a trade-off between conductivity and flexibility; its zero-dimensional structure limits sensitivity and multidirectional detection; and chemical inertness hinders functionalization for superhydrophobicity [202, 203].

##### Polymer Conductive Materials

Poly(3,4-ethylenedioxythiophene) (PEDOT) [204], polyaniline [205], and Ppy [206] represent several classic conductive polymers widely used in flexible electronics [207]. Their fundamental advantages include metal-like electrical conductivity (up to 10^3^–10^5^ S m^−1^) upon doping, while retaining polymer-specific flexibility, solution processability, and low density [208, 209].

**Fig. 6:**
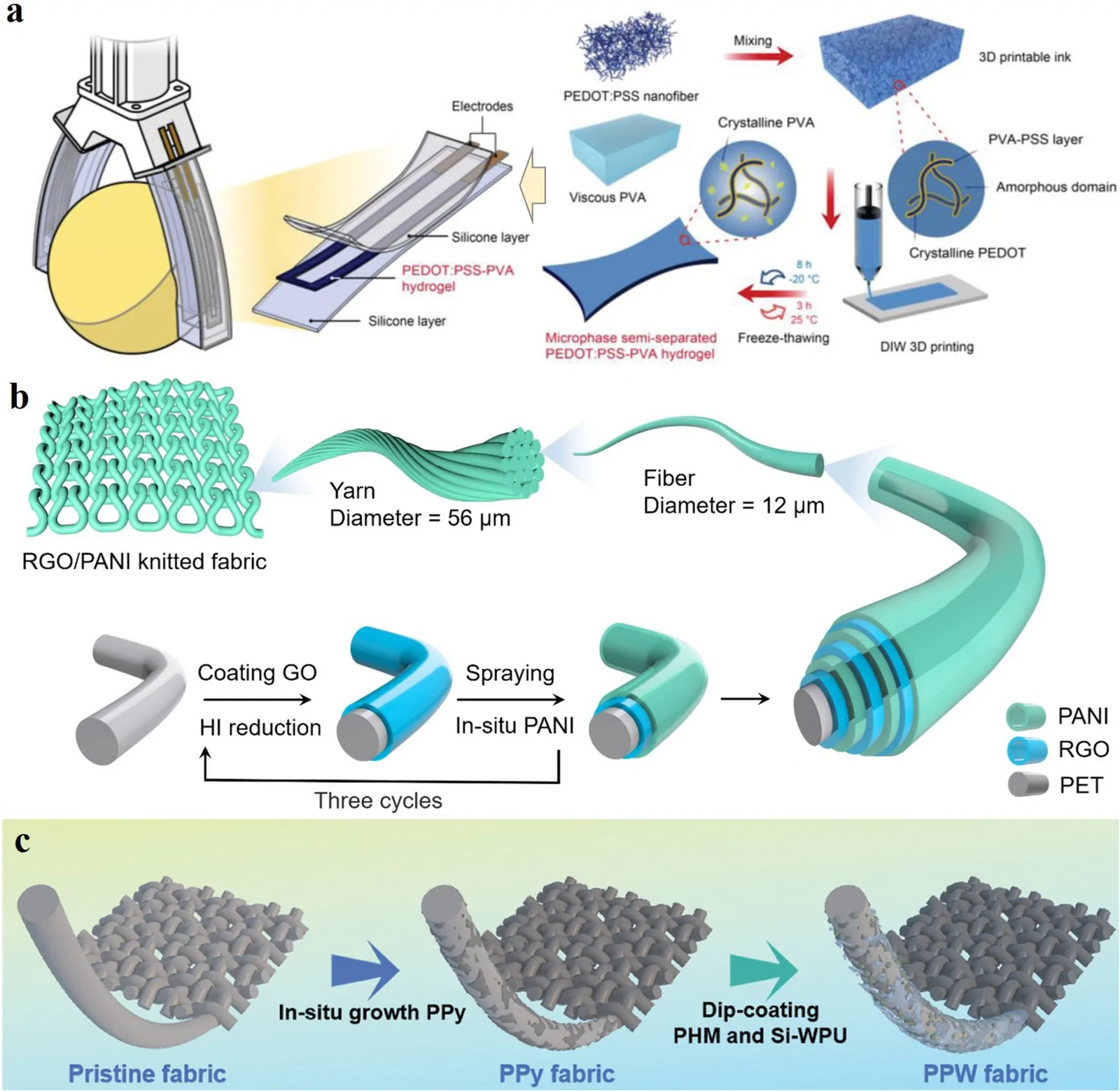
Polymer conductors: types, processing routes, and architectures. **a** 3D-printed, freeze–thawed conductive hydrogel patch [224]. Copyright 2022, John Wiley and Sons. **b** rGO/polyaniline laminate on textile via in situ polymerization [244]. Copyright 2024, Springer Nature. **c** Cotton fabric successively grafted with polypyrrole, dip-coated with polyaniline hollow spheres and amino-silane-modified polyurethane for wearable electronics [252]. Copyright 2024, John Wiley and Sons

PEDOT exhibits excellent molecular chain flexibility. When combined with ionic liquids or elastomeric polymers such as polyurethane, it forms intrinsically stretchable conductive networks that resist microcrack formation under strain, thereby maintaining conductive pathway integrity even at large deformations [210]. Among conductive polymers, PEDOT—particularly in its complex with poly (styrene sulfonate) (PEDOT:PSS)—achieves outstanding conductivity (exceeding 1000 S m^−1^ after optimization), enabling sensors with low initial resistance and high signal-to-noise ratio [211]. Its electrical conduction stems from a combination of electron transport and ion migration, and this mixed ionic–electronic character enhances strain sensitivity, facilitating high sensing performance [212]. PEDOT also demonstrates remarkable chemical and electrochemical robustness in ambient air, resisting oxidation and ensuring long-term operational reliability in complex environments. The intrinsic biocompatibility of PEDOT:PSS renders it a compelling candidate for wearable health monitors and implantable bioelectronics [213]. Furthermore, its commercial availability as an aqueous dispersion allows easy processing via various solution-based techniques [214–221] enabling straightforward integration with flexible substrates [222–227] for low-cost, large-scale manufacturing. For example, Shen et al. [224] fabricated a conductive polymer hydrogel strain sensor by combining PEDOT:PSS nanofibers with PVA via a unique microphase semi-separation network design, manufactured using 3D printing and successive freeze–thaw cycles (Fig. 6a). The resulting sensor exhibited both an ultrahigh fracture strain (300%) and negligible hysteresis. However, PEDOT:PSS suffers from several inherent drawbacks: the hydrophilic PSS causes moisture-induced signal drift [228, 229]; conductivity requires post-treatment and remains unstable; weak substrate adhesion leads to delamination; and it has limited stretchability, poor thermal stability, and mild acidity that may corrode the composite [164, 230].

Polyaniline achieves high electrical conductivity through protonic acid doping, a process that is both straightforward and low-cost. Its reversible, strain-dependent conductivity affords high-gauge-factor sensing, while inexpensive monomers and ambient-condition polymerization permit wafer-scale manufacturing. By adjusting synthesis parameters, diverse nanostructures can be obtained, enhancing both conductivity and interfacial adhesion. Moreover, polyaniline exhibits good chemical and thermal robustness, contributing to long-term sensor reliability [207, 231]. To overcome its intrinsic brittleness, polyaniline is often integrated with flexible substrates [232–235] via in situ polymerization [236–238], 3D printing [239, 240], or blending methods [241, 242]. Researchers also frequently combine polyaniline with other materials to construct multilayer or three-dimensional architectures for high-performance wearable strain sensors [243–247]. Hong et al. [244] fabricated an rGO/polyaniline electronic textile by alternately coating a knitted textile substrate with in situ rGO and in situ polymerized polyaniline, forming a laminated structure (Fig. 6b). The resulting e-textile exhibited outstanding air permeability and high sensitivity over a broad strain range (~ 0.0625%–200%). It also demonstrated excellent sensing durability, maintaining performance after severe mechanical deformation and routine machine washing. Nevertheless, polyaniline exhibits severe inherent brittleness, making it prone to cracking under mechanical deformation and necessitating composite modification to enhance flexibility. Its conductivity is highly dependent on acid doping and environmental pH, resulting in poor environmental robustness [248].

Polypyrrole is typically synthesized via straightforward chemical oxidation or electrochemical polymerization under mild conditions, achieving high electrical conductivity upon polymerization. Polypyrrole is widely recognized for its good biocompatibility, and its monomer exhibits significantly lower toxicity than aniline [207]. Through in situ chemical polymerization, polypyrrole can firmly adhere to the surfaces of various substrates and even penetrate into porous materials, forming robust interfacial bonds [249–252]. This strong adhesion helps minimize the risk of conductive layer delamination during deformation, thereby enhancing the sensor’s operational durability. By employing template-assisted or self-assembly synthesis, polypyrrole can be engineered into well-defined nanostructures such as nanoparticles, nanowires, or nanotubes. These architectures facilitate the construction of high-surface-area, efficient conductive networks, which significantly improve strain-responsive sensitivity [253–256]. Wang et al. [252] fabricated a flexible wearable device with multimodal sensing and electromagnetic wave absorption capabilities. Based on a cotton textile substrate, the device was prepared through in situ growth of polypyrrole, followed by dip coating with polyaniline hollow microspheres and amino-silane-modified polyurethane (Fig. 6c). The resulting sensor demonstrated rapid response and recovery times, along with high sensitivity. However, polypyrrole suffers from oxidative degradation, poor stability, insufficient adhesion causing delamination under strain, and biocompatibility risks from residual toxic monomers.

As two core components of wearable strain sensors, substrate materials and conductive materials collectively support and enhance sensor performance. These endows the sensor with stable and highly adaptable physical support. Currently, research on flexible wearable strain sensors is increasingly focusing on material hybridization, architectural refinement, and process integration (Table 1). The current experimental design focuses solely on the initial performance of the sensor (e.g., high sensitivity, wide strain range, and high conductivity), without incorporating strategies to maintain performance under long-term cyclic use or complex environmental conditions—such as antiaging of the conductive network, long-term interfacial adhesion, or fatigue resistance of the substrate. Significantly, biocompatibility and skin safety are key design criteria for wearable sensors, guided by standards such as ISO 10993. Hydrogels, Ecoflex, and PDMS are suitable for long-term wear (cytotoxicity < grade 1). Natural rubber requires antiallergy modification, and textile fibers should exceed 5 μm to prevent irritation. Carbon-based materials (MXene, graphene, CNTs) offer high biocompatibility. AgNPs need > 50 nm size and surface coating to keep Ag⁺ below 0.1 ppm. Conductive polymers must limit monomer residues to < 0.5%.

**Table 1:** Study on the performance of wearable strain sensors

Materials	Method	Structure	Strain range (%)	GF	Strain cycles	References
Graphene@PDMS	Chemical vapor deposition	Porous	187	1500	5000	[53]
CB/AgNW@Ecoflex	Emulsion blending		50		3000	[52]
CNTs@SEBS	Solution blending	Serrated groove	240	17.6	50% < 2000	[57]
RGO@NR	Solvent casting		226	870	10% 1000	[67]
CNTs/AgNWs@TPU	3D printing	Bionic	140	< 45665	20% 10000	[79]
rGO/CNTs@polyurethane fabric	Lamination	Multilayer	300	< 549.78	200% 2000	[101]
MXene@Nylon/polyurethane yarn	Electrospinning		263	~ 17	20% 10000	[108]
RGO@PDMS/Spandex Fabric	Dip coating	Sandwich	60		30% 4000	[144]
MXene@TPU	Dip coating		106	3.2 × 10^6^	20% 2,000	[159]
CNTs/AgNWs@TPU hydrogel	vacuum-assisted filtration	microcrack	171	1.1 × 10^5^	20% > 10,000	[186]
CB/MXene@PDMS yarn	Coaxial spinning	Core sheath coaxial	283.59	< 12.09	10% 1000	[200]
PEDOT/MWCNT@Polyester latex	In situ polymerization	Net	100	< 6.1	50% 2000	[211]
PEDOT:PSS@gel	Blending	Net	> 300	1.426	100% 60000	[213]
PANI@Chitosan microsphere colloid	In situ synthesis	Wrinkle	600	0.35 kPa^−1^	1 kPa 5000	[248]
PPy@Gelatin-based hydrogel	Template degradation		250	< 27.2	25% 500	[244]

### Design Fundamentals of Superhydrophobic Surfaces

**Fig. 7:**
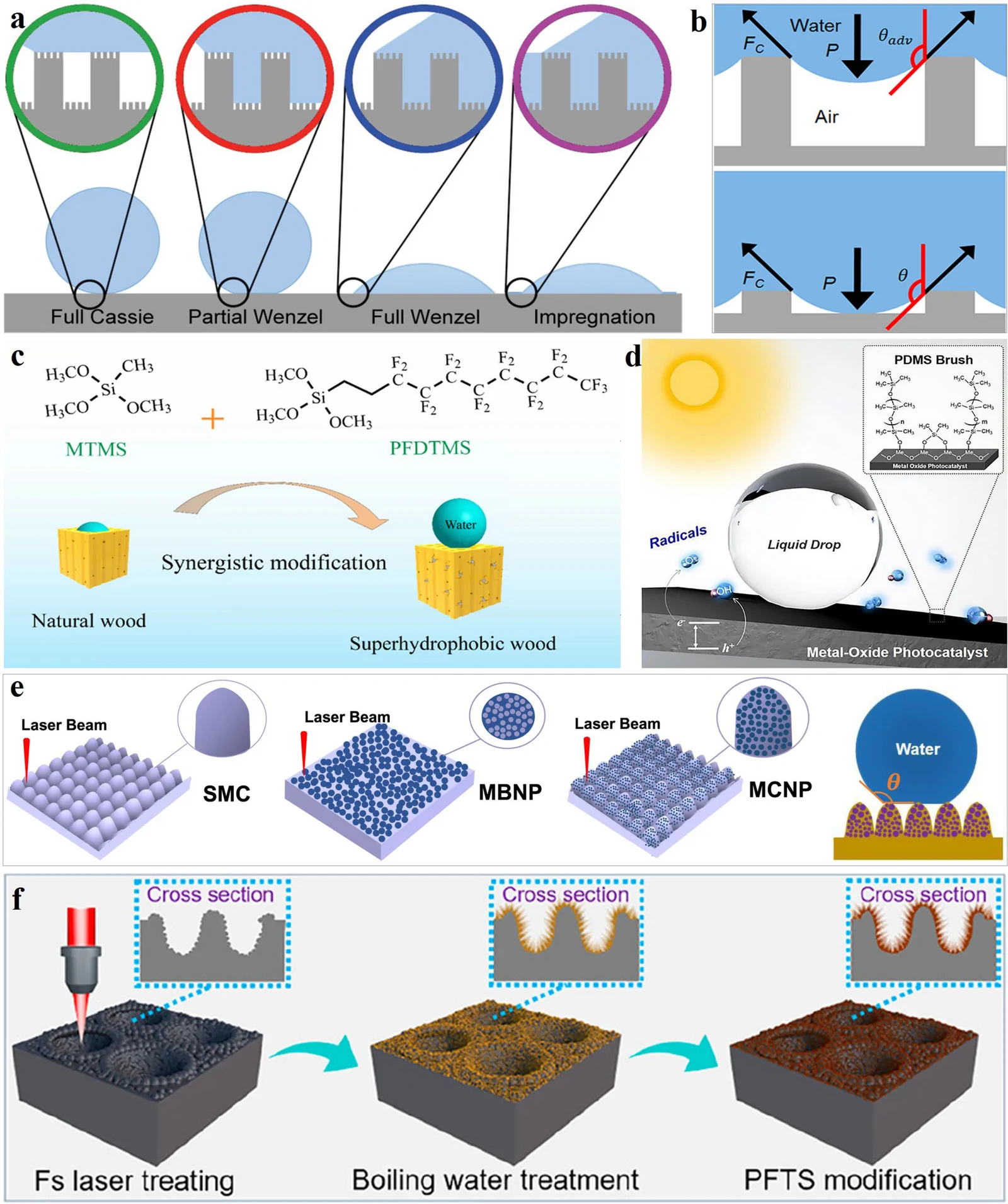
Mechanisms and structural designs of superhydrophobic surfaces. **a** Wetting regimes on a rough surface [263]. Copyright 2011, American Chemical Society. **b** Pressure-driven transition from the Cassie to the Wenzel state; sliding and sag are illustrated, with FC balancing the applied pressure P [264]. Copyright 2019, American Chemical Society. **c** One-step sol–gel deposition of fluorinated polymer and siloxane to render wood superhydrophobic [270]. Copyright 2024, American Chemical Society. **d** Fluorine-free polydimethylsiloxane superhydrophobic coating [274]. Copyright 2021, Elsevier. **e** Design of superhydrophobic surface structure to reduce liquid contact [276]. Copyright 2022, Springer Nature. **f** Periodic superhydrophobic arrays fabricated by femtosecond laser processing and boiling water treatment [282]. Copyright 2023, American Chemical Society

The introduction of superhydrophobic surfaces is regarded as a highly promising solution to the problems of water penetration and surface pollution. From the perspective of wetting theory, superhydrophobicity represents a special surface wetting behavior characterized quantitatively by the contact angle (*θ*_CA_) [257]. Solid surfaces are typically classified based on *θ*_CA_: Surfaces with *θ*_CA_ > 90° are considered hydrophobic, where water droplets tend to contract, but may still partially wet the surface [258]. When *θ*_CA_ exceeds 150° and the sliding angle (*θ*_SA_) is below 10°, the surface is defined as superhydrophobic [259, 260]. Such surfaces effectively repel water by maintaining a stable air–liquid interface, known as the Cassie–Baxter state [261, 262]. It should be noted that this state is not unconditionally stable. Increased internal droplet pressure—caused by impact, compression, elevated hydrostatic pressure, or reduced droplet size—may induce a transition from the Cassie to Wenzel wetting state. Additionally, if surface protrusions are insufficient in height, liquid may sag and contact the substrate (Fig. 7a, b) [263, 264].

The fabrication of superhydrophobic surfaces generally relies on the synergistic effect of chemical modification with low-surface-energy substances and physical micro-/nanohierarchical structures [265]. Low-surface-energy materials reduce the surface free energy, weakening liquid–solid intermolecular interactions and thereby suppressing wetting behavior [266]. Surface chemistry studies indicate that the surface free energy of a material is negatively correlated with its wettability—the lower the surface free energy, the greater the resistance to droplet wetting [267, 268]. Common low-surface-energy materials include fluorinated polymers, siloxanes, and long-chain alkanes [269]. Fluorinated polymers significantly reduce surface energy due to the high electronegativity of fluorine atoms and are widely used in superhydrophobic coatings (Fig. 7c) [270, 271]. Siloxanes form stable hydrophobic layers through their characteristic Si–O bond structures, exhibiting excellent water repellency [272, 273]. Long-chain alkanes create dense, low-energy barriers on material surfaces, blocking direct liquid–substrate contact and resisting wetting (Fig. 7d) [274]. In addition, by constructing micro-/nanoprotrusions and pores on the surface, an air layer can be trapped at the solid–liquid interface, forming a composite contact area that significantly reduces the actual liquid–solid contact area and enhances water repellency [275]. The core mechanism lies in the composite interface effect induced by the trapped air layer within the micro-/nanostructures (Fig. 7e) [276, 277]. When a droplet rests on such a surface, the interface is predominantly occupied by air, with the liquid only contacting the tips of the micro-/nanofeatures [278]. This minimized contact area prevents the droplet from spreading or adhering, resulting in easy roll-off and superior superhydrophobic performance [279].

The wetting behavior of a solid surface is governed by its surface energy and is further modulated by topographical roughness [280]. On smooth surfaces, the liquid–solid contact area is relatively large, and the contact angle is merely in the range of 90°–120°, thus failing to achieve superhydrophobicity. Conversely, in the absence of low surface energy, liquid droplets tend to infiltrate the rough micro-/nanostructures, resulting in the formation of the “Wenzel” state wherein the droplets exhibit strong adhesion to the substrate [281]. Thus, superhydrophobic repellency arises from low-surface-energy chemistry and micro-/nanoscale roughness (Fig. 7f) [282]. These two mechanisms jointly maintain the Cassie–Baxter state, thereby ensuring the surface exhibits excellent superhydrophobic performance [283]. Based on the above principles, superhydrophobic surfaces can play an important role in enhancing and protecting the performance of wearable strain sensors.

Low-surface-energy coatings can also impede the conductive network of the sensor. Specifically, coating molecules adsorb onto the surface of conductive particles to form an insulating layer, shifting electron transport from direct tunneling to indirect tunneling. This increases the initial resistance and reduces strain sensitivity [284]. Additionally, the coating lowers the surface energy of the substrate, weakening the van der Waals forces between particles and the substrate. This makes particles more prone to slippage under strain, disrupting the continuity of conductive pathways and thereby decreasing the GF [285]. Moreover, coatings exceeding 50 nm in thickness form a rigid shell that hinders the transmission of deformation to the internal conductive network. Nonuniform coatings, in turn, can induce localized stress concentration, exacerbating the fracture of conductive networks and leading to a decline in GF [286]. Furthermore, the micro- and nanoscale rough structures essential for superhydrophobicity can compromise sensing performance by dispersing strain-induced stress, which inhibits localized deformation of the conductive network and ultimately reduces the gauge factor [287]. Therefore, achieving a balance between superhydrophobic protection and sensitivity is essential for superhydrophobic strain sensors.

### Integration Routes for Superhydrophobic Strain Sensors

Integrating superhydrophobicity into strain sensors is highly significant and is typically achieved through a two-step strategy: fabricating the strain sensor, followed by applying a superhydrophobic coating or incorporating superhydrophobic materials into the sensor structure [24]. A key consideration is that the superhydrophobic treatment must not compromise the strain detection capability. The coating or integrated materials should be applied in a manner that preserves the flexibility and sensitivity of the sensor [288–290]. Developing wearable strain sensors with superhydrophobic properties requires the synergistic combination of sensing performance, low-surface-energy modification, and the construction of rough structures [291, 292]. However, an inherent contradiction exists between superhydrophobicity and sensing function: Conductive materials are generally hydrophilic, whereas hydrophobic materials tend to be electrical insulators [293, 294].

**Fig. 8:**
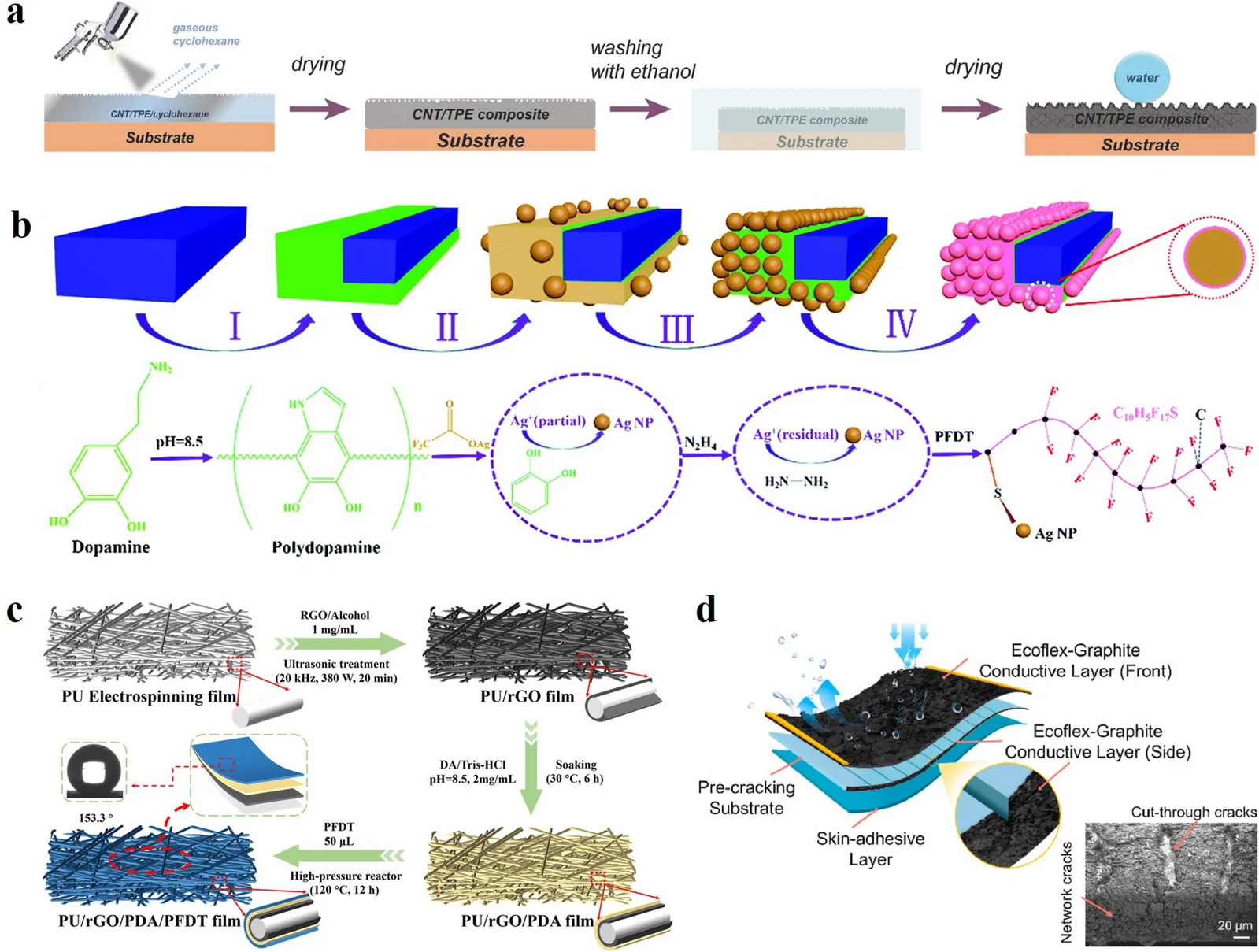
Integration routes to superhydrophobic wearable strain sensors. **a** Multiwalled carbon nanotubes (MWCNTs) spray-deposited onto a thermoplastic elastomer to form a multifunctional skin [296]. Copyright 2017, John Wiley and Sons. **b** Fluorinated AgNPs anchored to an elastic band via a polydopamine interlayer, yielding a bioinspired conductive rubber [28]. Copyright 2018, Royal Soc Chemistry. **c** Layer-by-layer stack of polyurethane/rGO/PDA capped with PFDT for a flexible, water-repellent strain gauge [300]. Copyright 2022, American Chemical Society. **d** 3D microcrack architecture in graphene/Ecoflex that couples high sensitivity with superhydrophobicity [303]. Copyright 2025, Elsevier

To resolve this conflict, we can adopt intrinsically hydrophobic conductive materials or fabricating conductive composites directly with superhydrophobic properties [295]. For example, Li et al. [296] sprayed multiwalled carbon nanotubes onto a thermoplastic elastomer solution to create a multifunctional smart coating, which imparted superhydrophobicity to the substrate while enabling real-time, omnidirectional monitoring of human motion (Fig. 8a). Hierarchical structural design to balance performance—first establishing a conductive network with nanomaterials, then performing surface hydrophobic treatment to simultaneously optimize both conductivity and superhydrophobicity [41]. Wang et al. [28] immobilized AgNPs on a rubber tape surface via a polydopamine layer and subsequently fluorinated the AgNPs, successfully producing a bioinspired conductive rubber composite that maintained both high electrical conductivity and superhydrophobicity (Fig. 8b).

The preparation methods of superhydrophobic wearable strain sensors fall into two primary categories. The first directly deposits conductive materials onto flexible substrates, followed by surface hydrophobic modification. Typical processes include layer-by-layer assembly, Mayer rod coating, drop casting, spray coating, spin coating, and laser direct writing, offering simplicity and cost-effectiveness [27, 297–299]. For example, Gao et al. [300] fabricated a multilayer superhydrophobic wearable strain sensor based on polyurethane/rGO/PDA/1H,1H,2H,2H-perfluorodecanethiol (PFDT), which combines high sensitivity, a wide working range, and good mechanical robustness while maintaining excellent sensing performance in high-humidity environments—meeting the demands of wearable devices in complex settings (Fig. 8c). The second category encapsulates conductive materials within flexible substrates, with hydrophobic modification applied to the substrate surface, often leveraging structural designs such as microcracks and wrinkles [301]. Mechanical hysteresis is critical for dynamic strain sensing but often overlooked. Superhydrophobic coatings can increase hysteresis via energy dissipation, reducing signal accuracy. Thicker, stiffer, or poorly bonded coatings worsen hysteresis; thin, compliant, well-adhered coatings minimize it [302]. Design must balance superhydrophobicity, sensitivity, and hysteresis for reliable performance.

Microcrack deformation induces significant changes in the resistance and current of conductive composites, enabling high sensitivity. Liu et al. [303] proposed a 3D crack structure: Through-cracks in the front conductive layer ensure high resistance variation, while side network cracks preserve sensing performance under large tensile strains to balance stretchability and sensitivity, mitigating low fidelity and high hysteresis (Fig. 8d). For graphene strain sensors, superhydrophobicity is achieved by attaching carbon black nanoparticles to an Ecoflex substrate to construct a rough structure, followed by polydimethylsiloxane modification. This imparts low surface energy even under 50% strain, endowing the sensor with excellent self-cleaning performance against various contaminants [304]. Other strategies include chemical etching, which enables precise control and material optimization for enhanced sensor performance. Specialized structural designs such as spider-like layered structure [305], gradient structure [306], and tile structure [307] are also widely used to achieve high-sensitivity detection of tiny mechanical motions.

### Multifunctional Integration and Key Performances

Benefiting from the repellency of superhydrophobic surfaces to water and moisture, the application scenarios of superhydrophobic wearable strain sensors have been significantly expanded compared to traditional counterparts [308]. Interfaces of conventional sensors are highly susceptible to adhesion of dirt, water droplets, or bacteria, leading to signal drift, performance degradation, and even biocompatibility risks—severely restricting their reliability and long-term robustness [309]. In contrast, superhydrophobic wearable strain sensors exhibit excellent antifouling and self-cleaning properties in air, breakthrough capabilities for underwater environmental monitoring, and crucial antibacterial functions, laying the foundation for the next generation of robust, reliable, and biosafe wearable electronic devices [310, 311]. This section systematically elaborates on the design principles, fabrication processes, and typical performances of these functionalized sensors, revealing how superhydrophobic surfaces transform “harsh environments” into “serviceable scenarios.”

#### Antifouling and Self-Cleaning

Conventional wearable strain sensors are prone to the adhesion of contaminants such as dust and oils in practical applications, which can cause signal drift, performance degradation, or even complete functional failure [312]. Inspired by the “lotus effect” in nature, researchers have endowed sensors with superhydrophobicity by constructing micro–nanohierarchical rough structures and modifying them with low-surface-energy substances [313]. This design results in an extremely high contact angle and a very low sliding angle for liquid contaminants, preventing them from adhering stably [314]. Under gravity or slight external force, these droplets roll off autonomously, thereby maintaining a clean and functionally stable sensing interface and achieving efficient self-cleaning and antifouling capabilities [315].

**Fig. 9:**
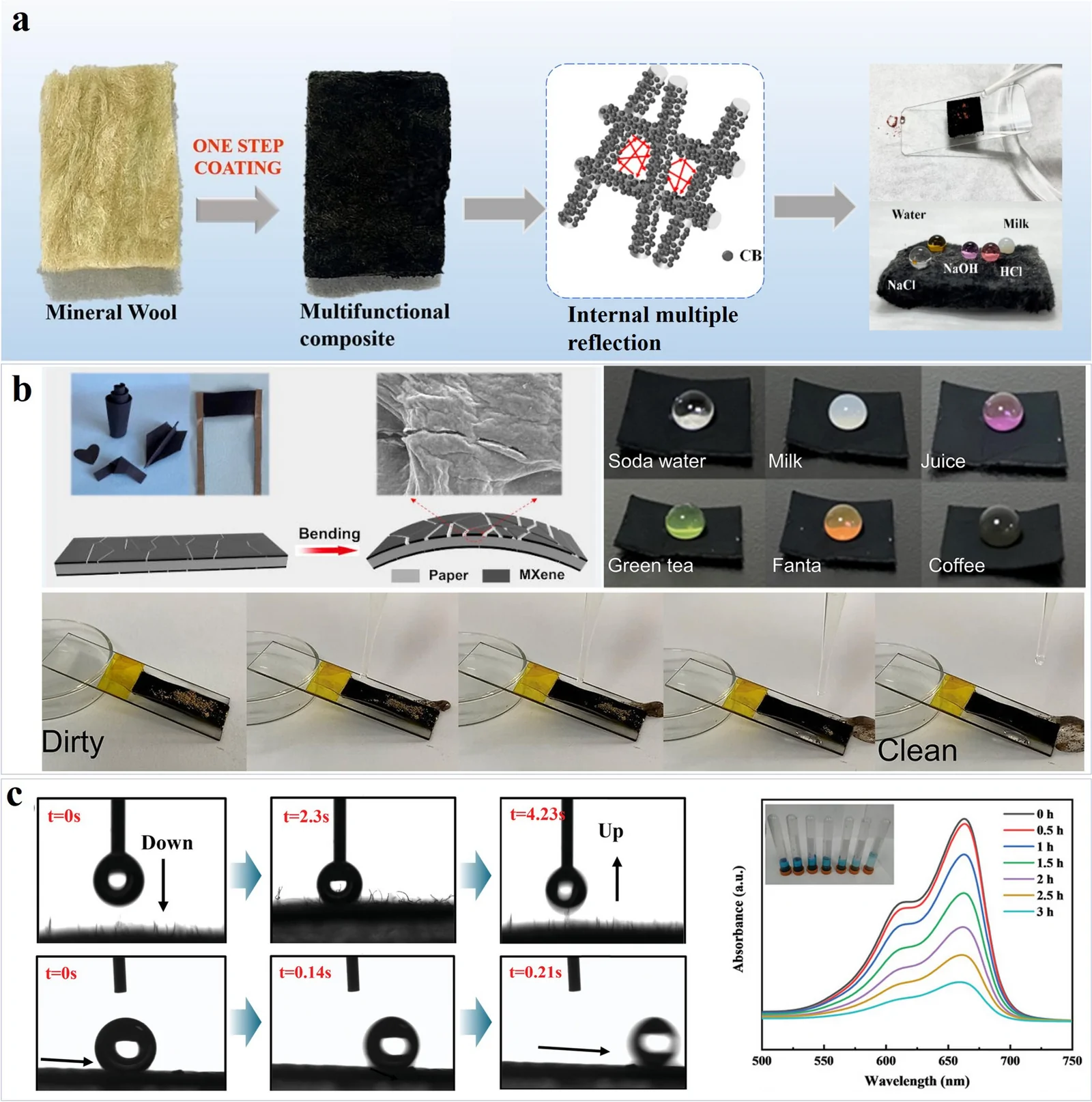
Antifouling performance of superhydrophobic wearable strain sensors. **a** Dip-coated waste mineral–wool/PDMS/carbon black composite converting solid refuse into a self-cleaning, stretchable gauge [318]. Copyright 2025, Elsevier. **b** Paper-based microcrack sensor: modulus mismatch between Ti_3_C_2_T_X_ MXene and cellulose generates high sensitivity; candle-soot over-layer provides > 150° water contact angle [320]. Copyright 2021, Elsevier. **c** MnO_2_-embedded fluoropolymer skin that couples super-repellence with photocatalytic degradation, restoring a contaminant-free surface under visible light [322]. Copyright 2023, John Wiley and Sons

To achieve compatibility between sensing functionality and self-cleaning performance, low-surface-energy materials such as epoxy oligomers are often compounded with conductive materials to construct rough structures for fabricating superhydrophobic strain sensors [316, 317]. For example, through a simple dip-coating process, carbon black nanoparticles and waste mineral wool are combined with PDMS to convert solid waste into multifunctional sensors with both superhydrophobicity and strain-sensing capabilities (Fig. 9a) [318]. Due to the modulus difference between materials, sodium carboxymethylcellulose is used as an interface enhancer to strengthen the interaction between materials, ensuring stable superhydrophobicity while improving interfacial bonding performance [319]. Alternatively, microcrack structures can be formed by leveraging the modulus difference between the conductive layer and the substrate. A superhydrophobic microcracked conductive paper-based strain sensor was prepared by dip-coating conductive Ti_3_C_2_T_X_ MXene onto printing paper, followed by depositing a superhydrophobic candle soot layer on its surface—achieving high sensitivity while the candle soot layer provides self-cleaning ability (Fig. 9b) [320]. After being immersed in various organic solvents or acid–alkali solutions for 6 h, the sensor still maintains stable superhydrophobicity and low surface adhesion, greatly broadening its application scope. Technically, oxidation and initiated chemical vapor deposition methods are commonly used; for example, PEDOT was applied via continuous vapor deposition to form a conformal polymer coating, fabricating superhydrophobic strain sensors [321]. To resist external pollution sources and achieve effective self-cleaning, the synergistic effect of superhydrophobicity and photocatalysis of MnO_2_ nanoparticles enables the degradation of organic contaminants, realizing rapid and efficient surface cleaning (Fig. 9c) [322]. These studies collectively converge on a core design philosophy: Through functional surface engineering, the synergistic integration of conductive sensing units and superhydrophobic protective layers endows sensors with the capability to resist complex liquid contaminant erosion, significantly enhancing their service life and reliability in harsh environments.

#### Underwater Sensing Signal Acquisition

Once conventional flexible wearable strain sensors are submerged in water, water molecules plasticize the polymer matrix, short-circuit conductive pathways, and shield strain signals, precluding underwater sensing and monitoring [323, 324]. The primary strategy for superhydrophobic flexible wearable strain sensors to overcome these issues is constructing micro-/nanoscale hierarchical roughness on the surface and grafting low-surface-energy molecules to form a Cassie–Baxter state air cushion, thereby achieving water-repellent effects [325]. Meanwhile, deformable conductive pathways such as cracks, island–bridge structures, porous foams, or fiber networks ensure the maintenance of a percolation network under stretching, bending, and compression [21, 326, 327]. Overall, by mimicking the skin’s “epidermis–dermis–hypodermis” multilayer structure, the hydrophobic layer, sensing layer, and stretchable substrate are sequentially coupled to realize “waterproofing without signal blocking” [328].

**Fig. 10:**
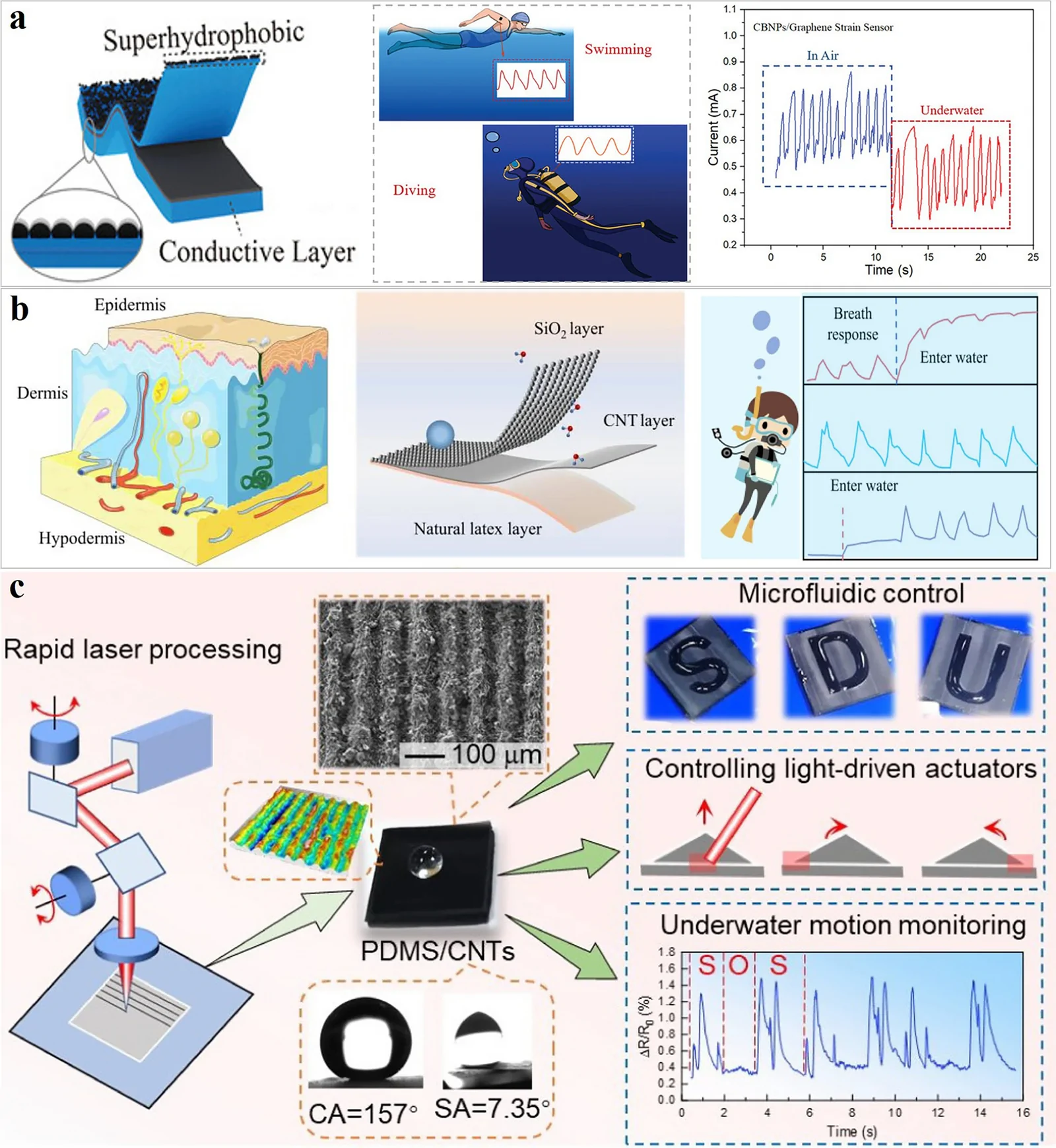
Sensing monitoring capability underwater of superhydrophobic wearable strain sensors. **a** Intrinsically superhydrophobic graphene/Ecoflex/carbon black film with periodic cracks: GF 653 at 90% strain and < 2% respiratory error underwater [304]. Copyright 2023, John Wiley and Sons. **b** Skin-mimetic MWCNTs/SiO_2_ bilayer that gives zero signal loss at 20 cm depth while tracking a diver’s motion [333]. Copyright 2024, Elsevier. **c** Laser-ablated PDMS/CNTs fiber (WCA 157° at 75% strain) detecting 0.5% bending underwater [335]. Copyright 2025, American Chemical Society

The realization of underwater sensing mainly falls into three categories of strategies. The first is the intrinsically superhydrophobic conductive framework: Conductive nanomaterials are blended with elastomers, followed by pre-stretching and releasing to form periodic cracks; subsequent spraying of fluorosilane or plasma grafting reduces the surface energy [329]. Thus, crack opening and closing during sensor stretching induce exponential changes in resistance, while the Cassie state air layer on the surface prevents water from infiltrating the cracks [330]. Duan et al. [304] fabricated a stretchable, superhydrophobic, and self-cleaning graphene strain sensor by attaching carbon black nanoparticles to an Ecoflex substrate to construct surface roughness and modifying it with polydimethylsiloxane to achieve low surface energy under tensile conditions (Fig. 10a). It exhibits a gauge factor as high as 653.4 under 90% stretching and an underwater breathing monitoring error of < 2%.

The second is multilayer heterogeneous encapsulation: blade coating or spin coating an adhesive layer, an ion-conductive layer, and a superhydrophobic self-cleaning layer, with interlayer connections via hydrogen bonds or dynamic covalent bonds [331]. During operation, the ion-conductive layer endows high capacitive/resistive response; the outer hydrophobic film blocks liquid leakage; and the adhesive layer enables reversible underwater adhesion–detachment at 22.4 °C [332]. Sun et al. [333] prepared a multilayer multifunctional sensor by sequentially constructing a sensing layer composed of MWCNTs and a hydrophobic protective layer composed of silica on the elastomer surface, mimicking the structure of human skin (Fig. 10b). The superhydrophobic surface ensures stable strain monitoring even underwater, allowing the multifunctional sensor to monitor divers’ physiological signals in real time throughout underwater activities with no signal attenuation within a depth of 20 cm.

The third is “line–plane” integrated weaving: Liquid metal is first impregnated and squeezed dry, then silver nanoparticles are electrolessly plated, and finally fluoroalkylsilane is sprayed to form micropapillae [334]; alternatively, laser ablation is used to etch unidirectional grooves on the surface of PDMS/CNTs composite fibers (Fig. 10c) [335]. Its working principle is that stretching induces changes in contact resistance between fibers; the groove structure promotes Cassie state water droplet rolling off while enhancing photothermal-driven self-floating [336]. The laser-etched PDMS/CNTs fibers exhibit a water contact angle (WCA) of 157° under 75% strain, an underwater finger bending detection limit of 0.5%. Thus, through biomimetic design [337], multilayer structures [338], innovative composite materials [291], and advanced manufacturing processes [339], superhydrophobic wearable strain sensors are capable of high-sensitivity underwater operation.

Based on the structural characteristics and working mechanisms, the three underwater sensing strategies exhibit distinct adaptability to hydrostatic pressure. Intrinsically superhydrophobic conductive frameworks rely on surface air entrapment, which is effective in shallow water but vulnerable to water intrusion under high pressure [304]. Multilayer heterogeneous encapsulation provides enhanced physical protection and better pressure tolerance through a dedicated hydrophobic barrier [340]. Line‑plane integrated weaving structures exhibit superior mechanical stability and interfacial robustness, suggesting greater potential for deeper underwater applications [335]. However, systematic and quantitative comparisons of water pressure resistance at different depths remain lacking, representing a critical challenge for future underwater sensing research.

#### Antibacterial and Biofouling Resistance

Wearable strain sensors adhere to the skin for extended periods in health monitoring, sports training, and postoperative care. Their surfaces are prone to forming a “sweat–bacteria” microenvironment, leading to signal drift, skin infections, and even device failure [341, 342]. In recent years, researchers have integrated superhydrophobic liquid-repellent properties with antibacterial functions to construct flexible sensing interfaces that combine waterproofing, antifouling, and antibacterial capabilities. The superhydrophobic surface forms a physical barrier via trapped air, preventing bacterial adhesion, while Ag^+^ penetrates the membrane to kill residual bacteria. Their synergistic effect achieves stable antibacterial performance, with a broad-spectrum bacteriostatic rate exceeding 99% [343].

**Fig. 11:**
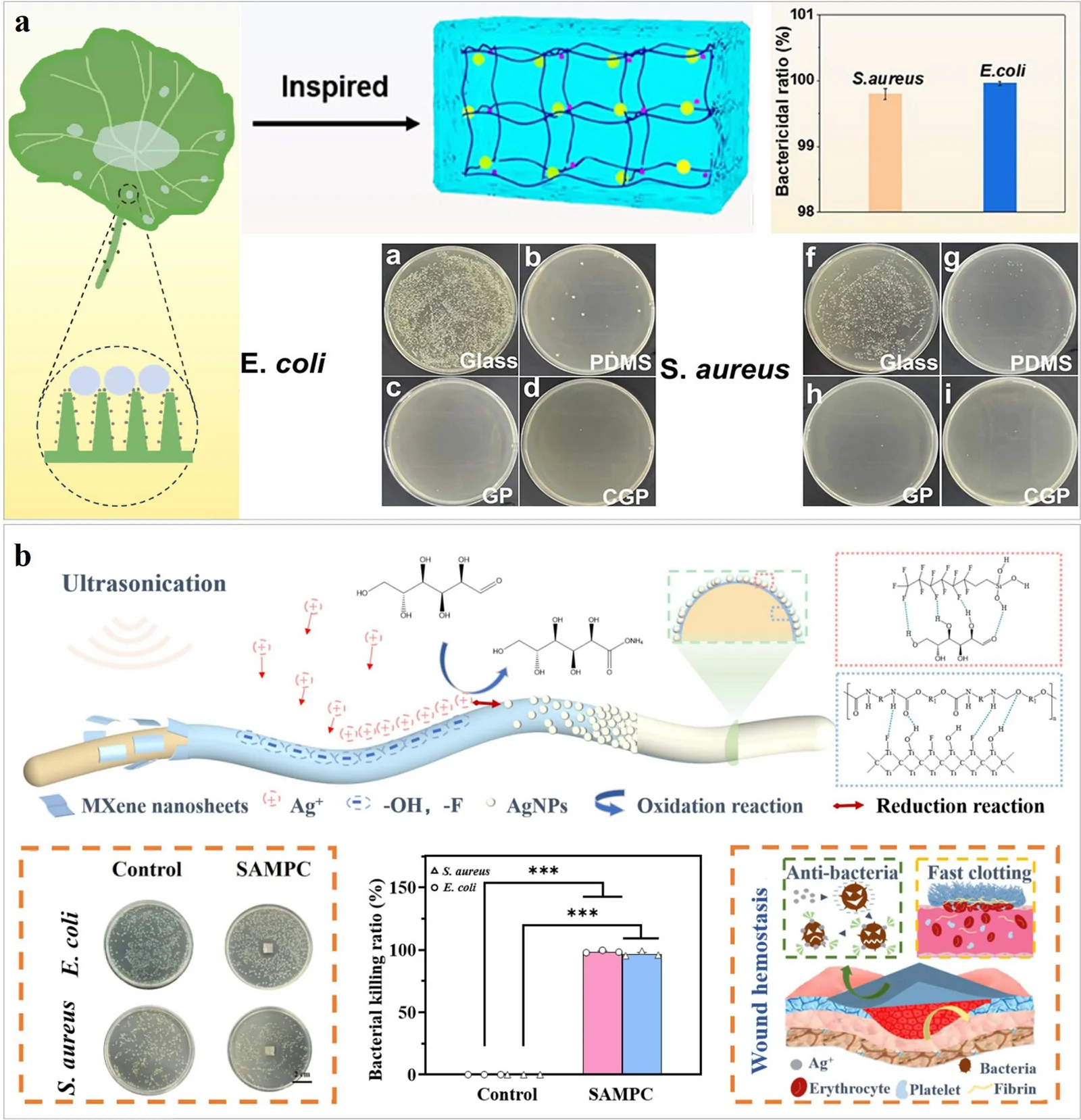
Antibacterial capability of superhydrophobic wearable strain sensors. **a** Lotus leaf-mimetic carbon black/PDMS film: Cassie air layer removes > 90% of bacteria under shear, yielding > 99% inhibition against *E*. *coli* and *S*. *aureus* [345]. Copyright 2023, American Chemical Society. **b** Multicore–shell AgNPs/MXene/polyurethane nanofiber web: 3,333 S cm^−1^ conductivity and 99.99% kill of drug-resistant E. coli after 7 d immersion in artificial sweat [348]. Copyright 2025, Elsevier

Current antibacterial research mainly adopts four strategies. First, hydrophobic low adhesion against bacterial colonization: The Cassie–Baxter state air cushion reduces the effective bacteria–surface contact area to < 10%, and shear water can remove > 90% of adherent bacteria [344]. Inspired by the superhydrophobic surface of lotus leaves, Hu et al. [345] fabricated a hydrophobic flexible antibacterial strain sensor based on carbon black/PDMS, with an antibacterial rate of over 99% against Escherichia coli and Staphylococcus aureus. Second, release-type metal nanobactericides: Ag^+^ and Cu^2+^ disrupt bacterial membrane potential and catalyze reactive oxygen species outburst (Fig. 11a). AgNPs and CuNPs are usually embedded on the surface of conductive networks via layer-by-layer spraying or supersonic cold spraying, followed by covering with a fluorosilane hydrophobic layer [343, 346]. Third, contact-type 2D materials/cationic polymers: This strategy relies on –OH/–F groups at the edges of Ti_3_C_2_T_X_ to physically puncture bacterial cell walls or the positive charge of quaternized polymers to electrostatically break down the membrane [347]. Xiao et al. [348] prepared flexible AgNPs/MXene/polyurethane nanofiber composites with a unique multicore–shell structure through MXene-induced in situ construction of superhydrophobic and conductive networks. It exhibits ultrahigh conductivity (up to 3333.0 S cm^−1^), a bacteriostatic rate of 99.99% against drug-resistant E. coli, and no bacterial proliferation in artificial sweat for 7 consecutive days (Fig. 11b). Fourth, multimode synergistic health regulation: Joule heating (40–45 °C) or the coupling of Ti_3_C_2_T_X_ with Ag^+^ release accelerates bactericidal kinetics; meanwhile, the superhydrophobic surface repels sweat to keep the skin dry [349]. To avoid “over-sterilization,” release-kill agents must control ion release (Ag^+^  < 0.05 ppm d^−1^) to ensure ≥ 99% antibacterial efficacy without disrupting skin flora, while contact-kill materials require optimized roughness (Ra < 1 μm) to prevent physical irritation. A core–shell AgNPs/MXene/polyurethane sensor achieves 99.99% antibacterial activity and a Draize score < 0.5 (ISO 10993-10), demonstrating synergy between antimicrobial function and skin safety [348].

The introduction of superhydrophobic interfaces has elevated wearable strain sensors from precision mechanical sensing elements to intelligent systems capable of actively adapting to and resisting harsh environments. Through surface and structural design, the sensors acquire lotus leaflike self-cleaning ability [308]. The air cushion layer on their surface can effectively block moisture, enabling underwater monitoring [350]. Simultaneously, the combination of superhydrophobic surfaces with antibacterial components constructs a dual defense line of “physical barrier and chemical killing,” significantly reducing the risk of infections and malfunctions caused by sweat and bacteria [351]. These advances establish superhydrophobic engineering as both a robust route to extend the environmental tolerance of sensing devices and a central pillar for realizing multifunctional integration, high reliability, and biosafety—thereby accelerating the scalable deployment of flexible electronics in real-world, high-complexity settings (Table 2).

**Table 2:** Overview of key features of superhydrophobic wearable strain sensors

Materials	Method	Structure	Strain range (%)	GF	CA	References
MXene/CNTs@Ecoflex	Layer-by-layer assembly	Bridge	100	89.72	150–160°	[21]
Carbon black@TPU	Molecular self-assembly	Porous	≤ 100	9.68–21.11	179°	[26]
AgNPs@Rubber band	Precursor and dip coating	Papillary	≥ 1000	1.0 × 10^7^	> 160°	[28]
MWCNTs@SEBS	Electrospinning and ultrasonication anchoring	Hierarchical network	130	12,172.46	> 155°	[30]
Graphene@PDMS/PEEK	Laser-induced graphene	Array	10	565	158°	[37]
PPy@Nano–microcollagen fibers	In situ polymerization,		10	56.4	> 155°	[38]
Acid-modified CNTs/AgNWs@TPU	Self-assembled	Net	38–100	1.36 × 10^5^	154°	[274]
rGO@Silk/polyurethane fabric	Dip coating	Hierarchical	100	2.4	155°	[301]
MWCNTs–carbon black@Hydrogels		3D wrinkled	100	8.48	160°	[318]
AgNPs/MXene@Polyurethane	Self-assembly/electrospun	Core–shell	30	< 917	152.3°	[334]

## Robustness Bottleneck: Failure Mechanisms and Mitigation Strategies

**Fig. 12:**
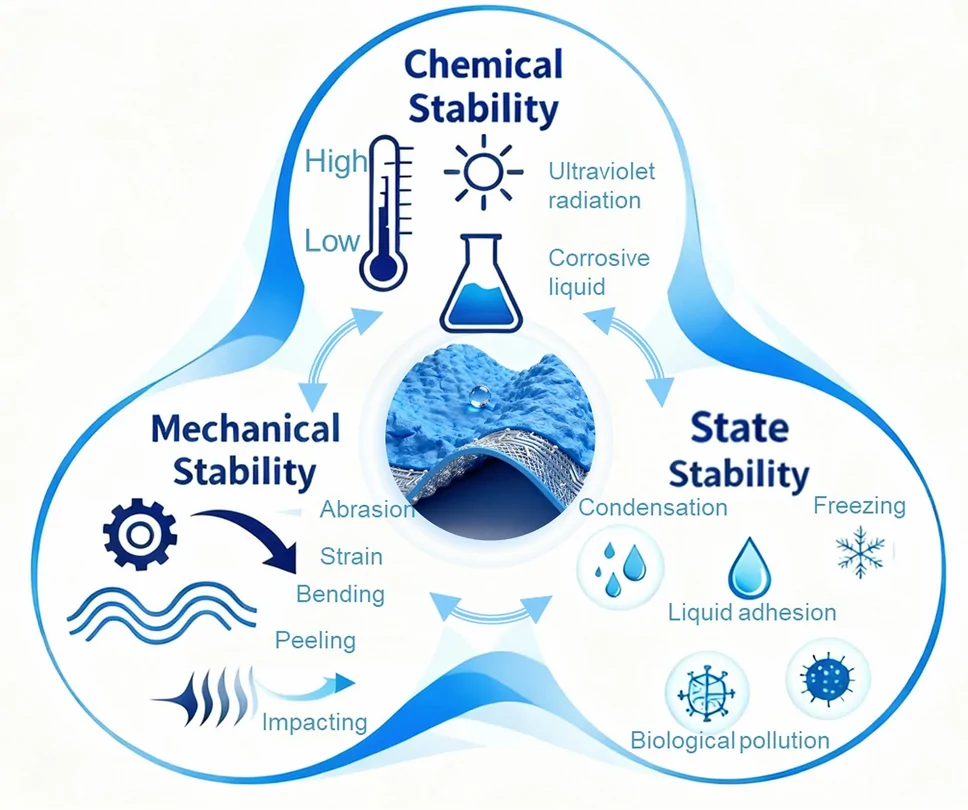
Interrelated challenges in chemical, mechanical, and state robustness of superhydrophobic wearable strain sensors

Despite the significant contributions of superhydrophobic flexible wearable strain sensors in enhancing the sensing robustness and functionality of flexible wearable electronics under complex environmental conditions, performance degradation in practical applications remains a major challenge [352]. Consequently, the environmental robustness of these sensors is of critical importance, encompassing chemical robustness [353], mechanical robustness [354], and state robustness (Fig. 12) [355]. Researchers have continuously sought to enhance robustness in harsh environments through optimized material combinations, fabrication methods, and surface structure design. This section establishes the robustness paradigm, offering a comprehensive overview of recent progress in environmental robustness, systematically examining testing and characterization methods for superhydrophobic durability, and exploring the underlying energy-based and force-induced failure mechanisms.

The robustness paradigm constitutes an integrated conceptual framework for designing, evaluating and optimizing superhydrophobic wearable strain sensors, targeting long-term stable operation under complex real-world conditions through unified conceptual foundations, design principles, and quantifiable metrics. Guided by a failure-centric perspective, this paradigm addresses critical sensor failure mechanisms under chemical, mechanical, and wetting stresses, pursuing enhanced chemical stability, mechanical durability, and wetting robustness.

### Chemical Destabilization Mechanisms and Countermeasures

Superhydrophobic wearable strain sensors must simultaneously repel water and survive harsh chemistry. Conventional dual-scale roughness topped with a low-surface-energy film fails when ultraviolet, aggressive ions or thermal spikes decompose the coating or delaminate the interface, a bottleneck that has stalled commercial rollout [356–358].

#### Corrosive Medium Attack

**Fig. 13:**
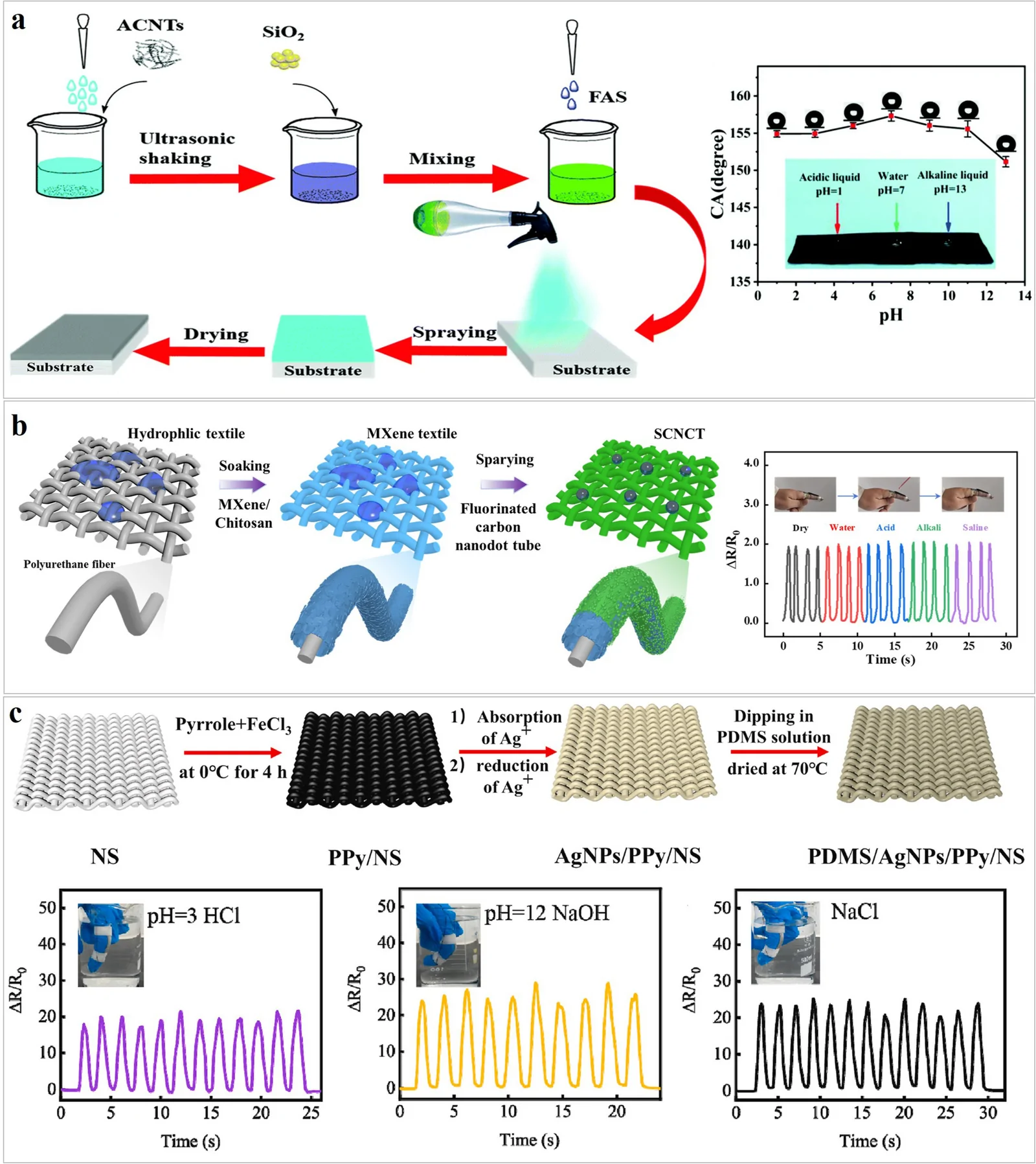
Resistance to corrosive medium attack. **a** Schematic of the spray-up route to a hierarchical fluorinated carbon nanotubes/SiO_2_ nanoshell that locks Cassie roughness against acid/base attack [297]. Copyright 2019, Royal Soc Chemistry. **b** Carbon–nanodot/CNTs/MXene microfiber fabric: illustration of the 2D glue dip-coating process and steady ∆R/R_0_ during 0.1 M HCl/NaOH exposure [362]. Copyright 2025, Elsevier. **c** PDMS/AgNP/polypyrrole/nylon strap: fabrication diagram and stable sensor output recorded in corrosive electrolyte [367]. Copyright 2022, Elsevier

The achievement of superhydrophobicity is highly dependent on the elaborately constructed micro-/nanostructures on the sensor surface. These structures form unique topological morphologies at the microscale, enabling stable gas–liquid interfaces that prevent liquid wetting [359]. However, when sensors are long-term exposed to strong acidic (pH 1) or alkaline (pH 14) solutions, the micro-/nanostructures face severe erosion challenges [360]. Researchers have fabricated superhydrophobic conductive flexible composites by immobilizing AgNPs on elastic substrates via PDA or chemical deposition, followed by further immersion in PFDT solution [298]. The nanostructures formed by AgNPs and their stable chemical properties endow the material with excellent hydrophobicity in acidic, alkaline, and saline environments. Nevertheless, the rough structures composed solely of AgNPs struggle to protect sensors from corrosive environments during service. To address this, Gao et al. [297] developed a corrosion-resistant superhydrophobic sensor by spray coating a hierarchical fluorinated carbon nanotube/SiO_2_ nanoparticle structure on elastic tapes, which demonstrated reliable operation under corrosive liquid interference (Fig. 13a) [361]. Our group designed a superhydrophobic carbon nanotube/MXene/microfiber composite fabric by dip coating a 2D conductive adhesive layer, followed by spraying a fluorinated 0D/1D conductive network suspension onto the substrate (Fig. 13b) [362]. This fabric retained superior superhydrophobicity after 3 h of immersion in 0.1 M strong acid/alkali and 3.5 wt% saline solution, while maintaining stable sensing performance in these corrosive media. Studies have shown that PDMS modification on the surface of flexible sensors can provide excellent corrosion resistance [363, 364].

To meet the comfort requirements of wearable strain sensors and enhance their sensing robustness in corrosive environments, numerous studies have constructed composite conductive structures on elastic fabrics combined with PDMS modification [365, 366]. For example, Peng et al. [367] built an AgNPs/polypyrrole composite conductive network on elastic nylon strips, followed by coating a thin protective PDMS layer, successfully fabricating a highly sensitive and superhydrophobic strain sensor based on PDMS/AgNPs/polypyrrole/nylon strips (Fig. 13c). This sensor exhibited stable operation and reliable output signals in acidic, alkaline, and saline solutions. The primary strategy to improve corrosion resistance involves combining composite conductive materials with polymer protective layers [368]. For example, constructing conductive networks via PDA template assistance, fabricating fluorinated nanostructures through spray/dip coating, or integrating PDMS surface modification can significantly enhance the hydrophobic robustness and signal reliability of sensors in acid, alkali, and saline solutions [352]. Such architectures simultaneously guarantee reliable sensor operation under corrosive conditions while preserving wear comfort and mechanical compliance. However, real-world sweat–sebum mixtures (ISO 3160-2) pose greater risks to superhydrophobic coatings than single pH solutions. Electrolytes and lactic acid accelerate corrosion, while lipids disrupt Cassie–Baxter states and weaken adhesion. Lipid adsorption reduces interfacial strength, promoting delamination under strain. Wet–dry cycles induce swelling–shrinkage effects and microcracks—dynamic static pH tests cannot replicate. Thus, artificial sweat–sebum tests are essential for real-world validation [369].

#### UV Irradiation-Induced Degradation

With environmental pollution and the gradual thinning of the ozone layer, the intensity of outdoor UV radiation has increased progressively, posing challenges to the chemical robustness of superhydrophobic sensors under UV exposure. UV radiation possesses high energy, and its irradiation on superhydrophobic wearable strain sensors triggers a series of complex chemical reactions, with photocatalytic degradation being the most critical one [370]. Si–O bonds in organosilicon coatings—critical for maintaining the coating’s structure and performance—are prone to cleavage under UV light, compromising the sensor’s long-term serviceability.

**Fig. 14:**
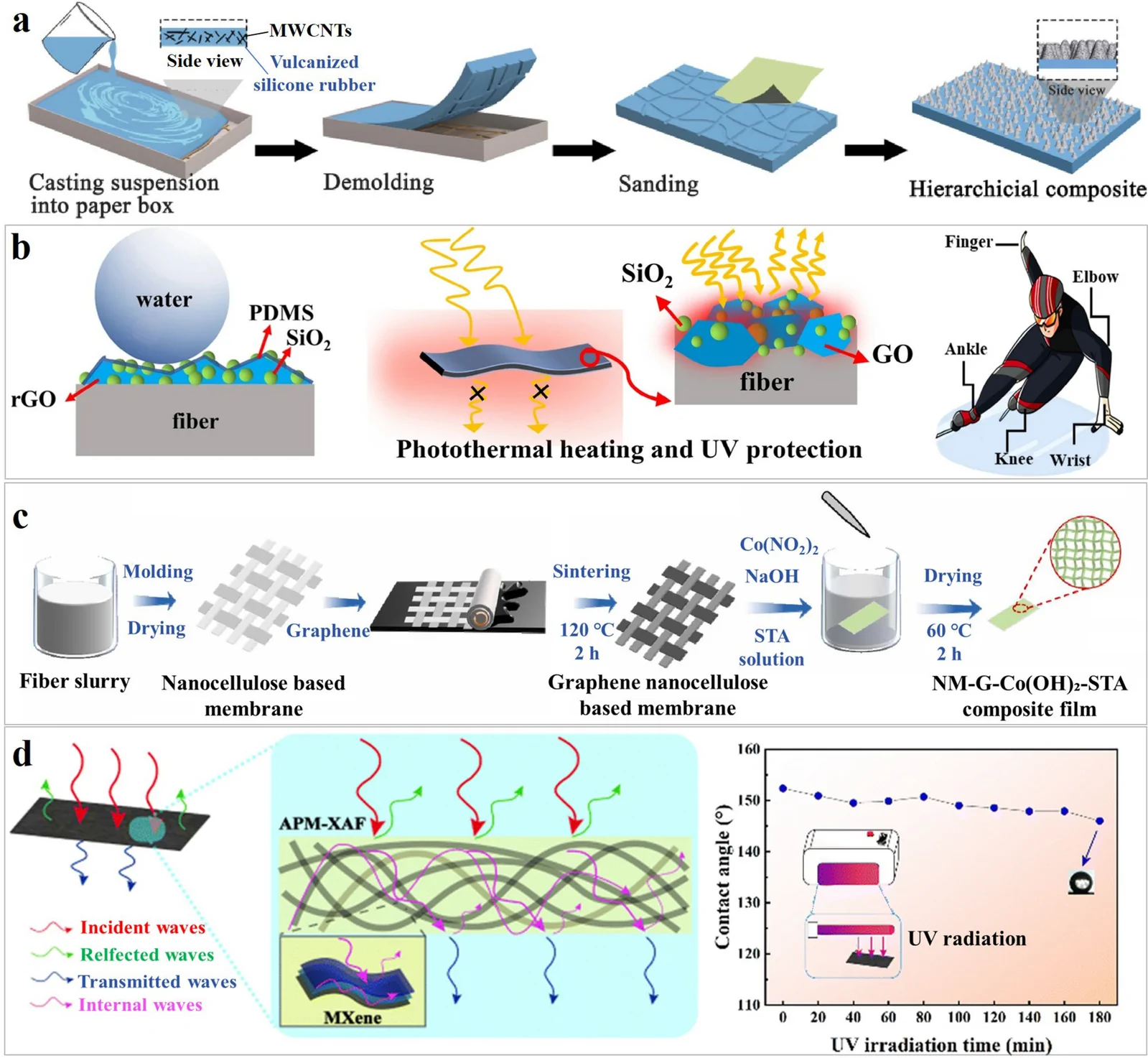
Resistance to UV radiation-induced degradation. **a** Schematics of the coating-free route to intrinsically superhydrophobic sensing films [371]. Copyright 2021, Elsevier. **b** @rGO/SiO_2_–PDMS textile: water-repellent profile and UV shielding/photothermal defense [145]. Copyright 2022, Springer Nature. **c** Fabrication diagram of nanocellulose/graphene/Co(OH)_2_/stearic acid superhydrophobic strain sensor [372]. Copyright 2023, Elsevier. **d** MXene-based electronic textile interacting with UV light and its contact angle evolution under irradiation [373]. Copyright 2025, Royal Soc Chemistry

To address photocatalytic degradation, multiple material and functional integration strategies have been developed for UV resistance of superhydrophobic wearable strain sensors, mainly by incorporating UV-shielding or photostable components to enhance UV tolerance. Jia et al. [371] pioneered the integration of a conductive layer, a superhydrophobic layer, and a stretchable polymer into a single system, achieving a durable coating-free superhydrophobic sensor (Fig. 14a). Vinyl methyl silicone rubber matrices resist photo-oxidative degradation induced by UV, ozone, and oxygen, retaining high sensitivity across a broad strain window under prolonged solar exposure—an optimal combination for kinematic monitoring in harsh outdoor environments.

Lu et al. [145] constructed a superhydrophobic sensor by electrostatic self-assembly of rGO on chitosan fabrics, followed by dip coating with SiO_2_ nanoparticles and PDMS (Fig. 14b). The sensor demonstrates excellent UV protection and photothermal effects, applicable for long-term outdoor wear. Meanwhile, Li et al. [372] prepared a nanocellulose/graphene/Co(OH)_2_/stearic acid composite film via blasting and dip coating. Through the synergistic effect of Co(OH)_2_ and graphene, a highly conductive hierarchical structure was formed, achieving an ultralarge contact angle of 166°. Importantly, graphene’s ability to absorb and dissipate UV light, combined with Co(OH)_2_’s UV reflection and scattering capabilities, endows the sensor with effective resistance to UV degradation (Fig. 14c). Recently, Feng et al. [373] have fabricated a multifunctional electronic textile based on airlaid paper via ultrasonic welding and dip coating, integrating PDA/MXene/aminopropyltriethoxysilane-1H,1H,2H,2H-perfluorooctyltriethoxysilane. This sensor possesses excellent breathability and superhydrophobicity and maintains stable strain responses under ultrasonic cleaning, high temperatures, and UV irradiation, indicating high structural durability and functional integrity in UV environments (Fig. 14d).

#### Extreme Temperature Environments

Superhydrophobic wearable strain sensors typically consist of a superhydrophobic coating, conductive materials, and flexible substrate [374]. Their structure may be damaged under high-temperature environments due to mismatched coefficients of thermal expansion among different materials. Moreover, superhydrophobic coatings and substrates tend to decompose at elevated temperatures, leading to performance degradation [320]. Thus, the chemical robustness of superhydrophobic strain sensors under high temperatures is particularly critical.

**Fig. 15:**
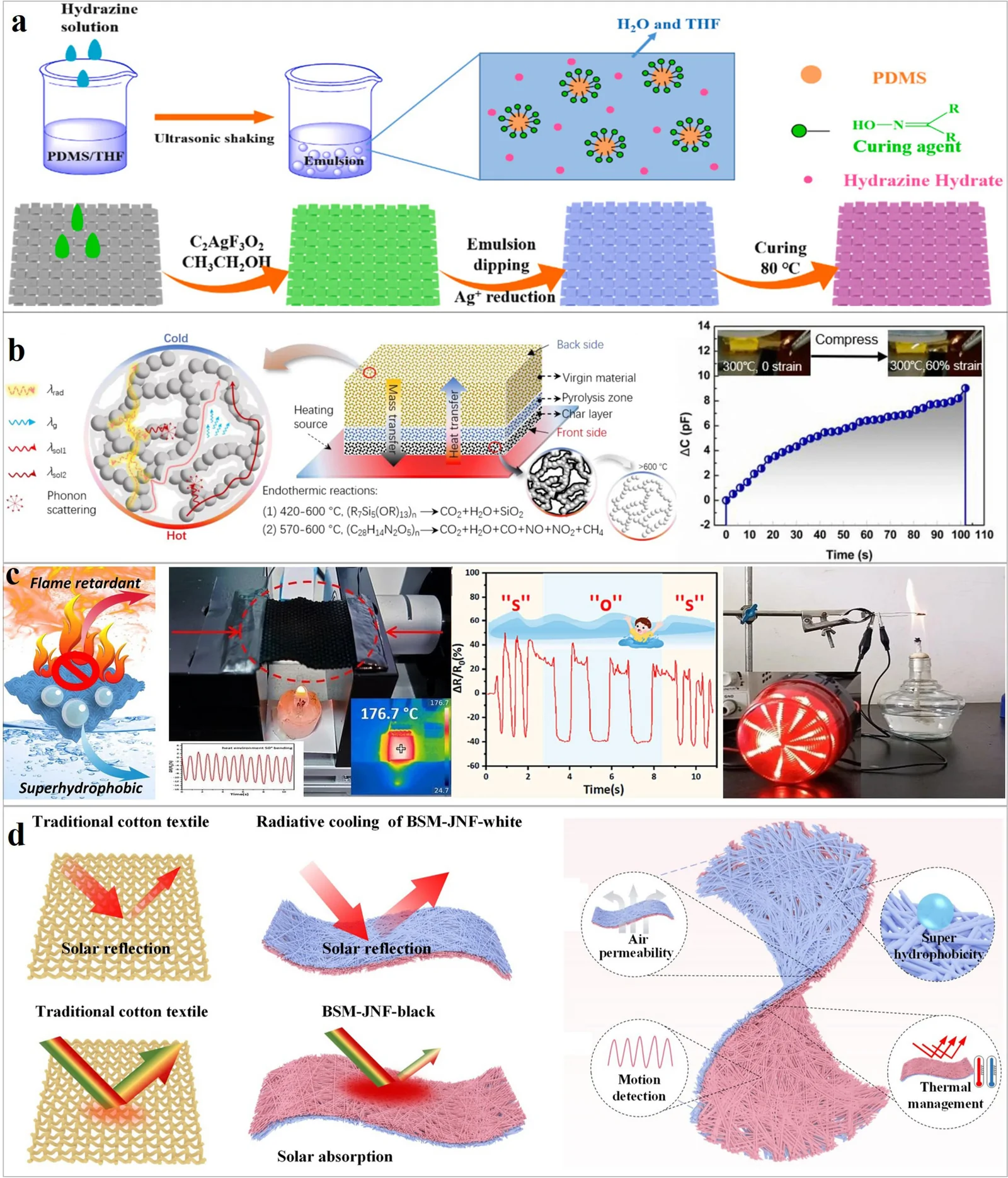
Resistance to extremely high-temperature environments. **a** Schematic of the emulsion-dip route to a porous AgNPs@PDMS conductor that preserves σ and θ_CA_ after exposure at 120 °C [377]. Copyright 2021, Elsevier. **b** Polyimide/silica aerogel with a “slice/sphere” dual morphology: architecture and superhydrophobic strain response retained at 300 °C [378]. Copyright 2022, Elsevier. **c** Breathable flame-retardant fabric sensor: thermal durability and 1 s fire alarm signaling at 176.7 °C [379]. Copyright 2024, Elsevier. **d** Janus TPU/PDMS/MWCNTs/AgNWs nanofiber membrane: asymmetric heat-spreading/insulating functions that stabilize strain output under thermal cycling [380]. Copyright 2025, Elsevier

Current strategies for imparting high-temperature robustness converge on the synergistic integration of thermally stable polymers, inorganic nanomaterials, and conductive fillers with hierarchical micro-/nanoscale architectures and surface functionalization. These approaches enable sensors to maintain mechanical robustness, hydrophobicity retention, and signal robustness under high-temperature conditions [375, 376]. Wu et al. [377] constructed a porous AgNPs@PDMS conductive coating via emulsion impregnation. In situ reduced AgNPs form a percolating network in PDMS, endowing the coating with high electrical conductivity and excellent Joule heating effect (Fig. 15a). Notably, the coating retains unchanged contact angle and conductivity after heat treatment at 120 °C for 10 h, indicating that the synergistic effect of PDMS encapsulation and AgNPs effectively inhibits high-temperature oxidation and structural degradation.

Regarding hierarchical architecture design, Huang et al. [378] developed a polyimide/silicone superhydrophobic strain sensor through rational “slice/sphere” dual-morphology design and a two-step gelation process (Fig. 15b). While maintaining superhydrophobicity and high porosity, the sensor achieves 90% weight retention up to 474 °C and retains strain responsiveness at 300 °C. Its high-temperature robustness stems from the combined effect of the heat-resistant polyimide framework and the excellent thermal insulation of the gel. Additionally, Liu et al. [379] modified cotton fabric to successfully fabricate a flexible, breathable fabric-based strain sensor with ultrahigh moisture resistance, flame retardancy, and environmental durability (Fig. 15c). At high temperatures (~ 176.7 °C), the sensor exhibits stable and reliable response signals to 50° bending forces while demonstrating superior thermal insulation. It can effectively transmit “SOS” Morse Code signals from drowning victims for water rescue and act as a fire alarm sensor with rapid response during fires.

Recently, Zong et al. [380] constructed a TPU/PDMS/MWCNTs/AgNWs Janus nanofibrous membrane via blend electrospinning (Fig. 15d). This membrane integrates superhydrophobicity with dual-mode thermal regulation and delivers strain signal composite, and the Janus structure effectively suppresses interface failure caused by coefficient of thermal expansion mismatches, enhancing sensing reliability in high-temperature environments [312, 381]. So current research on high-temperature tolerance of superhydrophobic wearable sensors can be categorized into three main strategies: (i) incorporating thermally stable segments or flame-retardant components into polymer matrices [382, 383]; (ii) exploitation of inorganic refractories as conductive and/or protective layers [357]; and (iii) hierarchical micro-/nanoarchitectures that suppress thermal degradation, limit heat ingress, and reinforce interfacial cohesion [356]. Collectively, these approaches substantially extend sensor operability in extreme thermal environments. Current studies only improve high-temperature resistance through material blending and structural design, but fail to solve the inherent thermal expansion mismatch and interface failure issues under long-term or ultrahigh temperatures, and lack systematic evaluation under coupled high-temperature and mechanical deformation conditions.

Under low-temperature environments, cross-linked networks become more brittle with diminished elasticity. When interfacial stress exceeds the adhesion between the coating and substrate, coating cracking or delamination is induced [384]. Additionally, some coating materials such as wax-based hydrophobic agents may undergo crystallization at low temperatures. Wax-based hydrophobic agents typically have low melting points; at low temperatures, their molecules transform from a disordered liquid or semisolid state to an ordered solid crystal [385]. This phase transition causes changes in material volume and morphology, damaging the originally regular and ordered micro-/nanostructures.

**Fig. 16:**
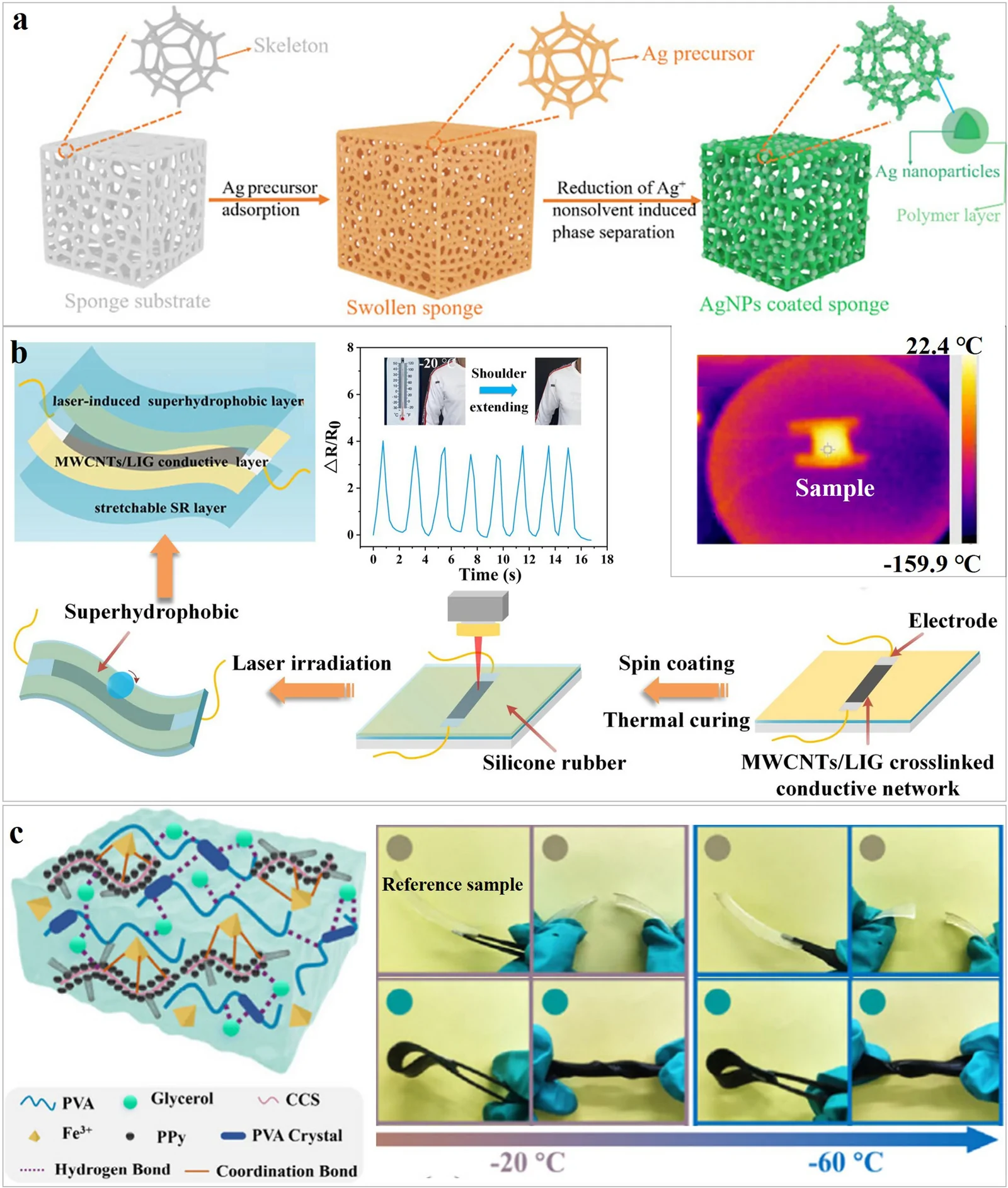
Resistance to extremely low-temperature environments. **a** Self-derived superhydrophobic AgNP@rubber sponge foam: fabrication diagram and Joule heating profile at –20 °C [387]. Copyright 2020, American Chemical Society. **b** SR/MWCNTs/LIG/SR composite sensor: preparation schematic and stable strain curves recorded under freezing conditions [388]. Copyright 2022, Elsevier. **c** PVA/CCS/glycerol/FeCl_3_/polypyrrole/MWCNTs antifreeze hydrogel: architecture and tensile performance at sub-zero temperatures [389]. Copyright 2024, American Chemical Society

To enable reliable application of superhydrophobic wearable strain sensors in cold or freezing environments, numerous studies have effectively enhanced their low-temperature robustness through material selection, structural design, and functional integration [386]. A study successfully fabricated a self-derived superhydrophobic composite foam by adsorbing silver precursors on rubber sponges, reducing them to AgNPs, and constructing a porous high-roughness surface via nonsolvent-induced phase separation technology [387]. The material exhibits high-performance strain and pressure sensing concurrently with pronounced Joule heating and photothermal responses, enabling autonomous thermal management that preserves sensor functionality at sub-ambient temperatures (Fig. 16a). These electrothermal and photothermal pathways thus constitute robust, on-device strategies for low-temperature operation (Fig. 16b) [388]. A key trade-off exists between coating thickness and heat transfer in photothermal/electrothermal deicing systems. Thicker coatings enhance mechanical durability, but introduce thermal resistance due to low-conductivity polymers, reducing deicing efficiency. Designs must balance thickness for robustness with minimal thermal resistance for rapid deicing—a critical challenge for active deicing superhydrophobic sensors.

Addressing the key issue of hydrogel freezing at low temperatures, Liu et al. [389] developed a multi-cross-linked hydrogel tolerant to ultralow temperatures of –60 °C by constructing a double-network matrix and incorporating various conductive fillers and freely mobile ions (Fig. 16c). On this basis, a superhydrophobic Ecoflex layer (WCA ~ 159.1°) was deposited via spray coating and laser engraving, which effectively isolates external moisture and reduces ice crystal adhesion. This enables the sensor to maintain stable performance in low-temperature and underwater environments, successfully applied in diving gesture recognition. Antifreeze components such as glycerol and ionic liquids are mainly distributed inside the hydrogel matrix instead of the sensor surface. These internal additives thus hardly affect the superhydrophobic performance (contact angle and sliding angle), which is determined by an independent external coating. A layered structure is the optimal strategy for collaborative optimization: The inner hydrogel provides antifreeze properties, while the outer superhydrophobic layer maintains stable surface wettability. In response to environmental temperature changes, a strain sensor with superamphiphilicity and photothermal conversion capability was constructed based on nanomicron collagen fibers (polypyrrole/SCB@PP-CFs) via in situ polymerization and spray impregnation [38, 390].

The enhancement of chemical robustness in superhydrophobic wearable strain sensors primarily relies on the synergistic optimization of materials and structures. Current strategies include: utilizing inorganic nanomaterials to construct corrosion-resistant micro-/nanoframeworks combined with low-surface-energy modification via fluorinated polymers [391]; and employing hierarchical composite structures and interface enhancement technologies to retard corrosion penetration and coating delamination [392]. For UV irradiation, shielding components such as graphene and metal hydroxides are incorporated to inhibit polymer photodegradation through light absorption and scattering [145, 357]. In high- or low-temperature environments, heat-resistant segments, flame-retardant components, thermal insulation fillers, and active thermal management are adopted to maintain performance robustness [312, 383].

Despite promising advances, translating superhydrophobic strain sensors into field-ready devices is still impeded by a triad of intertwined limitations: Laboratory durability assays rarely extend beyond brief, single-chemical exposures and therefore leave unresolved the synergistic assault of complex, real-world electrolytes coupled with years of dynamic flexing [30]; the superhydrophobic skin itself adheres only weakly to compliant substrates, inviting delamination whenever temperature excursions or swelling–shrinking cycles accumulate strain at the interface [393]; and the very nanoparticles or fluorinated grafts that confer UV, thermal, or chemical resistance simultaneously stiffen, seal, or irritate the underlying textile, breaching the flexibility, breathability, and biocompatibility thresholds that wearable electronics must satisfy to remain unobtrusive [394].

Establishing unambiguous chemical robustness criteria is pivotal for field deployment of superhydrophobic wearable strain sensors: After 100 h of UV irradiance, pH 1–14 immersions, 3.5 wt% NaCl and − 20 to 200 °C thermal cycles, the water contact angle must decrease by < 10°, hysteresis must shift by < 5°, the electrical response must drift by < 15%, and no visible peeling, discoloration or cracking may appear. Only when this level of chemical tolerance is achieved without sacrificing mechanical compliance or wearer comfort can these sensors transition from laboratory curiosities to reliable tools in health care, environmental monitoring, and specialized industry.

### Mechanical Failure and Reinforcement

In extremely harsh and dynamically varying real-world application scenarios, the structural robustness challenges faced by superhydrophobic wearable strain sensors represent a core bottleneck hindering their functional accuracy and long-term robustness. Unlike chemical robustness, which primarily addresses static or slowly changing chemical environments, structural robustness directly relates to the durability and reliability of sensors under dynamic, repeated mechanical stresses [290]. Their functional core is highly prone to failures such as crack propagation, interfacial delamination, and structural collapse under mechanical deformation, leading to signal distortion or even permanent functional loss [324, 371]. Thus, how to coordinate the unification of “strain sensing” and “superhydrophobicity” under dynamic environments through ingenious mechanical structural design has become a focus of recent research in this field. This review will highlight the latest strategies in this direction, including biomimetic structural design, multilevel interfacial adhesion, intrinsic stretchable network construction, and the application of dynamically reversible material systems, aiming to explore fundamental approaches to reinforce device structures for achieving performance robustness of superhydrophobic strain sensors under long-term, complex mechanical service conditions.

The challenge of mechanical robustness lies in the irreversible disruption of the conductive network under cyclic strain, which precipitates a permanent loss of conductivity [323], coupled with abrasive wear or collapse of the micro-/nanoscale roughness that obliterates superhydrophobicity [395]. High strain beyond a critical threshold increases micro-/nanostructure spacing, reducing breakthrough pressure and inducing Cassie–Wenzel transition—even without coating damage. This critical strain defines the limit for maintaining superhydrophobicity; below it, structures recover elastically; above it, liquid infiltration causes irreversible failure [396]. Thus, designing structures to maximize critical strain is key to preserving superhydrophobicity under large deformation. This is further complicated by the intrinsic trade-off between low modulus for large-strain deformation and high cohesive energy for mechanical robustness in molecular design. Low-modulus substrates, with limited cross-linking and surface hardness, are prone to microstructural damage under friction or stretching, leading to loss of superhydrophobicity [31]. Conversely, strengthening interfacial adhesion—via PDA coating or plasma treatment—increases interchain interactions, raising modulus and reducing elongation, thus compromising flexibility [354]. In recent years, research focus has shifted from mere function realization to robust mechanical structural design, with strategies primarily categorized into the following three types.

#### Dynamic Deformation Fatigue

The key to eliminating irreversible damage in the conductive network under repeated deformation is to embed covalent cross-links or strong interfacial bonding within a three-dimensional architecture that intrinsically endows the composite with extensibility and toughness, enabling large-strain cycling without structural failure [397, 398]. The key is to effectively avoid or mitigate the initiation and propagation of cracks within the conductive network during deformation via optimized interfacial engineering, thereby maintaining stable electrical properties and superhydrophobic functionality [399].

**Fig. 17:**
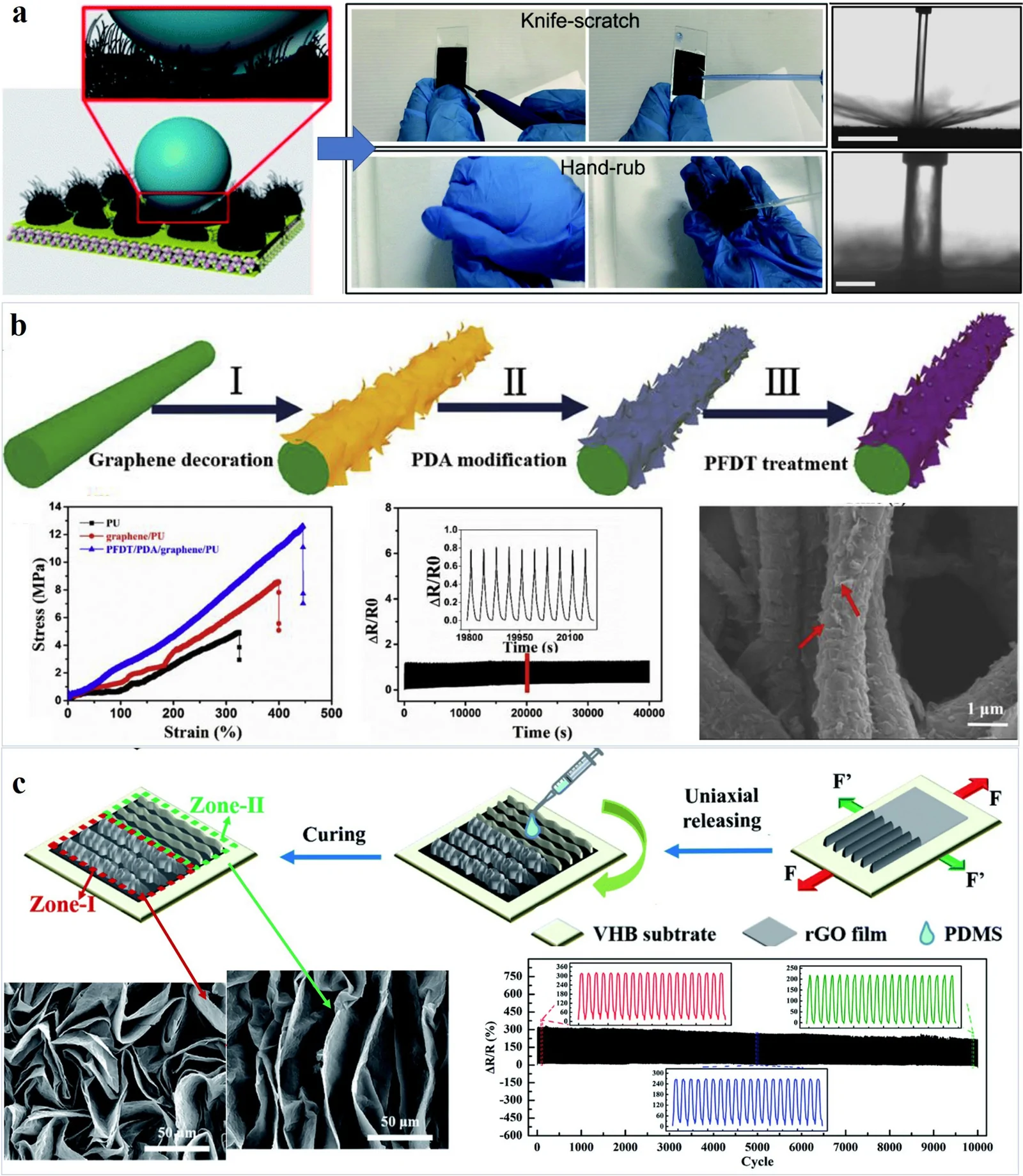
Resistance to dynamic deformation and structural fatigue. **a** Schematic surface architecture and robustness of a superhydrophobic strain sensor enabled by perfluorinated modification [376]. Copyright 2020, Royal Soc Chemistry. **b** Fabrication protocol and robustness of a PFDT/PDA/graphene/polyurethane nanofiber composite yielding flexible, superhydrophobic conductivity [402]. Copyright 2022, Royal Soc Chemistry. **c** Preparation principle, surface morphology, and cyclic tensile response of a gradient wrinkle rGO/PDMS strain sensor [405]. Copyright 2021, Royal Soc Chemistry

The primary approach to achieve this goal is the intimate compositing of conductive nanomaterials with high-performance elastomers to form uniform and tough intrinsic stretchable systems [400]. Wang et al. [376] modified PDMS matrices via a perfluorination strategy and composited them with multiwalled carbon nanotubes, significantly enhancing the material’s liquid impalement resistance without compromising mechanical robustness (Fig. 17a). This sensor can tolerate strains up to 200%, and its superhydrophobicity and electrical properties show no significant degradation after various mechanical damages (e.g., hand rubbing, sandpaper abrasion) and high-speed fluid impact. For flexible substrates such as textiles or nanofibers, interfacial modification is crucial for improving structural robustness. PDA is widely used as an efficient interfacial modifier due to its excellent adhesive properties [401]. PDA modification markedly strengthens both π–π bridging between graphene sheets and interfacial adhesion to TPU fibers. This strong interfacial interaction effectively transfers mechanical stress from the relatively rigid conductive layer to the more ductile polymer substrate, thereby avoiding interfacial delamination and significantly improving the overall mechanical properties of the composite, including Young's modulus, tensile strength, and elongation at break (Fig. 17b) [402].

Mechanical robustness is gauged through a standardized suite of tests: Monotonic uniaxial stretch to large strain defines intrinsic extensibility and ultimate strength [403], while cyclic tension/compression fatigue quantifies durability, demanding that the electrical response remains virtually unchanged after thousands to tens of thousands of strain cycles [404]. For example, Chu et al. [405] developed a gradient wrinkled strain sensor based on rGO/PDMS, which demonstrated outstanding fatigue resistance, with no significant decay in electrical signal output after 10,500 cycles (Fig. 17c). Collectively, these mechanical test data confirm that the strategy of designing intrinsically stretchable and tough networks enables the fabrication of superhydrophobic strain sensors that combine high stretchability, high sensitivity, and excellent mechanical durability. In high-frequency dynamic sensing, time-dependent recovery is critical yet often overlooked. Beyond structural damage, high-frequency fatigue can cause temporary superhydrophobicity loss via surface functional group reorientation—even with intact microstructures [406]. This reversible degradation requires recovery time for hydrophobic chains to reorient outward. Surfaces stable under static conditions may fail transiently under high-frequency fatigue, necessitating consideration of recovery kinetics for reliable wearable applications.

#### Wear and Peel Resistance

Inspired by the microstructures of natural surfaces, constructing multiscale hierarchical rough structures is regarded as a key strategy for simultaneously achieving superhydrophobicity and excellent mechanical robustness [323]. The core concept lies in providing effective physical protection for the fragile internal conductive functional layer and the surface superhydrophobic micro–nanostructures through sophisticated macro- and microstructural design [305]. These architectures redistribute mechanical loads via controlled structural deformation, yielding coatings and devices with exceptional durability under complex tribo-mechanical stimuli—including friction, tension, and flexure [339].

**Fig. 18:**
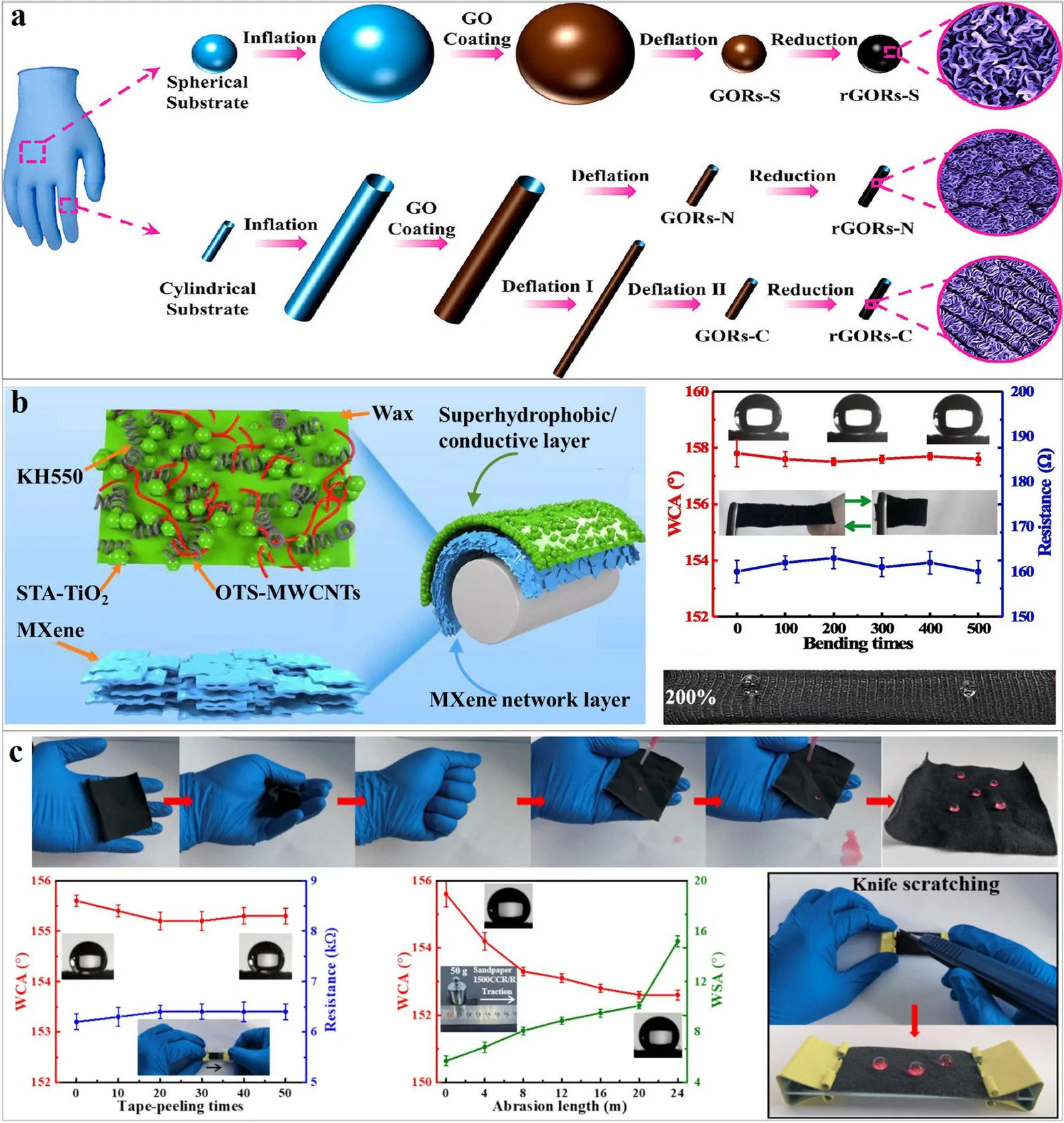
Resistance to abrasive wear and interfacial peeling. **a** Schematic of the rGOR-based wearable strain sensor fabricated by a 4D shrink–wrap process [407]. Copyright 2019, American Chemical Society. **b** Structural schematic diagram and robustness testing of synergistic coupling dual conductive coating-based electronic textiles [334]. Copyright 2023, Elsevier. **c** Mechanical durable superhydrophobic fabric strain sensor based on dual conductive layer [299]. Copyright 2025, Elsevier

Wrinkle/buckling structures are formed by depositing a conductive layer on a pre-stretched elastic substrate and then releasing the strain to generate controllable wrinkle or buckling patterns [398]. These structures can gradually flatten rather than fracture directly upon stretching, thereby extending the sensing range and protecting the integrity of the conductive layer. An anisotropic rGO buckling pattern (rGOR) was generated using a dimension-controlled 4D shrinkage method (Fig. 18a) [407]. The resulting flexible rGOR-based strain sensor can detect both large and subtle human motions, achieving high sensitivity and ultrahigh areal stretchability (up to 2690%). It also demonstrated excellent durability in human motion monitoring, resisting hand rubbing, ultrasonic cleaning, and machine washing. Similarly, gradient microwrinkle structures of PDMS/MXene/rGO have been fabricated via oblique filtration and pre-stretching techniques [408]. These studies leverage the same principle to unify high sensitivity with a wide strain range.

Core–shell/multilayer protective structures involve constructing functionally layered systems in which a robust outer layer shields the inner sensitive conductive layer. A representative design is the dual conductive layer/coating strategy. For example, a superhydrophobic shell comprising hierarchical papillae over an MXene conductor shields the conductive core from oxidative and aqueous attack while arresting crack propagation by mechanical decoupling [334]. MXene–CNTs bridge structure further utilizes a bridging effect to reduce stress concentration, leading to more gradual crack distribution and a synergistic improvement in linear range and sensitivity (Fig. 18b) [21]. Similarly, constructing a PDA interfacial layer, an ACNTs/Cu functional layer, and PDMS encapsulation on textiles has realized excellent chemical and mechanical durability through such a hierarchical synergistic strategy [401]. The robustness of these designs is evaluated using tests that simulate complex mechanical wear in practical applications. These mainly include abrasion tests (e.g., sandpaper friction [403], tape peeling [409]), bending/twisting cyclic tests [410], ultrasonication [33], and high-speed fluid impact tests [24]. Our group established a fabric-based composite with dual conductive networks, which maintained its superhydrophobic surface after dynamic stretching, bending and twisting cycles, sandpaper abrasion, tape peeling, water jet impact, and washing and drying (Fig. 18c) [299]. The composite exhibited stable electrical signals throughout 5000 stretching cycles and after bending and twisting, demonstrating the structural robustness of the design.

#### Self-Healing Capabilities

**Fig. 19:**
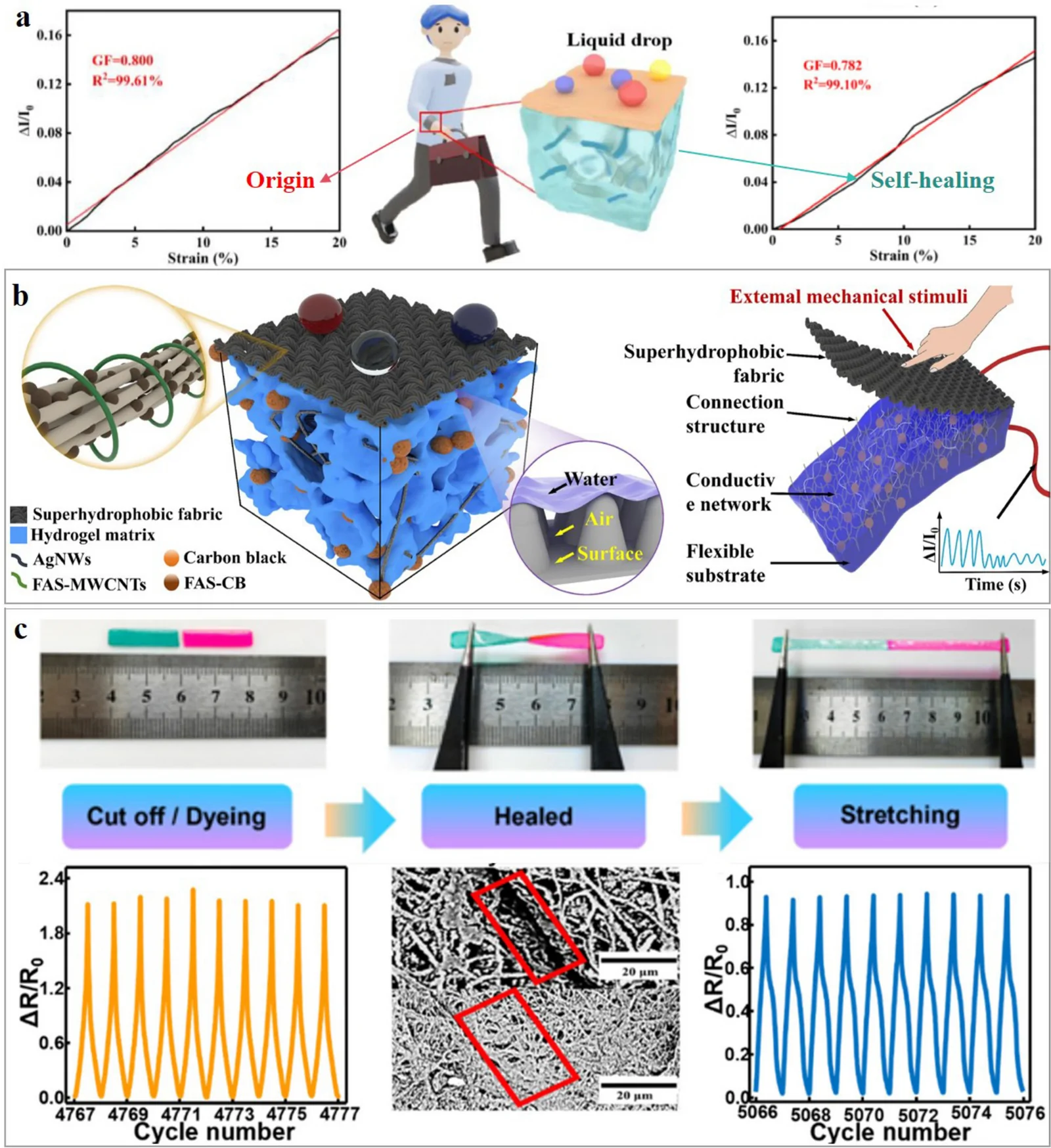
Resistance to macroscopic cutting and segmentation. **a** Wearable synergistic strain sensor created by overlaying a superhydrophobic layer on an electrically double-cross-linked hydrogel [411]. Copyright 2024, Elsevier. **b** Hydrogel–textile composite sensor that interlocks a polydopamine microcapsule-reinforced poly(vinyl alcohol)-poly(acrylic acid) hydrogel [414]. Copyright 2025, Elsevier. **c** Intrinsically self-healing, superhydrophobic electronic textile strain sensor fabricated by depositing rGO on an electrospun nanofiber mat [415]. Copyright 2023, American Chemical Society

The dominant strategy against macroscopic damage is to embed a self-healing architecture that spontaneously restores microtexture, superhydrophobicity, and sensing function after mechanical or chemical insult, thereby intrinsically extending service life and ensuring reliability in complex environments [302]. To this end, our group converges on implanting dynamic, reversible bonds whose scission–reformation closes damage pathways (Fig. 19a) [411], while concurrently exploiting segmental and surface mobility of hydrophobic chains that autonomously enrich low-energy moieties over wounded areas, instantly reinstating water repellency. In terms of material construction strategies, common methods include synthesizing self-healing polymer matrices with abundant dynamic bonds [412]. Intrinsic self-healing systems are constructed by designing polyurethane elastomers with numerous hydrogen bonds, or introducing disulfide bonds (with reversible exchange properties) into polymer cross-linking networks. On this basis, conductive fillers and low-surface-energy substances are uniformly dispersed in self-healing polymer precursors via blending, followed by curing to form composites integrating conductivity, superhydrophobicity, and self-healing capability [413]. Additionally, a stepwise construction strategy can be adopted—depositing a superhydrophobic functional layer on the surface of preformed elastomers containing dynamic bonds via spraying or dip coating to fabricate hierarchical self-healing sensors [412].

For performance evaluation, researchers typically quantitatively verify self-healing efficiency and structural robustness by systematically comparing changes in water contact angle, sliding angle, and sensing signal robustness before and after repair, as well as before and after mechanical treatments. Our group fabricated a superhydrophobic strain sensor with rapid and multicycle self-healing capability; after each damage event and subsequent self-recovery, its sensitivity, mechanical integrity, and superhydrophobicity remain unaltered (Fig. 19b) [414] And Gao et al. [415] developed an integrated sensor with superhydrophobicity and self-healing capability (Fig. 19c). It had a GF of 145 at 100% strain and a WCA of 153.6°, maintaining robustness over 10,000 tensile cycles at 50% strain, and could monitor human motions during swimming. This demonstrates the effectiveness and application potential of self-healing strategies in addressing structural robustness issues of superhydrophobic strain sensors during long-term service.

A key yet underexplored issue in self-healing superhydrophobic sensors is the time lag between conductivity and superhydrophobicity recovery. Conductivity restores rapidly via crack closure, while superhydrophobicity requires slower migration of low-surface-energy molecules, leaving wetting resistance temporarily compromised [412]. Future designs should synchronize both recovery kinetics through mobile hydrophobic components or hierarchical structures enabling simultaneous physical and chemical self-healing. A key challenge in self-healing superhydrophobic sensors is the limited long-term underwater stability of healed interfaces. Structural defects—such as weak chain entanglement, uneven hydrophobic component distribution, and poor adhesion—compromise durability [416]. Most sensors degrade upon prolonged immersion due to water infiltration, causing re-cracking and delamination. Current evidence insufficiently supports long-term reliability, highlighting healed interface defects as a critical research bottleneck.

Among various mechanical reinforcement strategies, wrinkled structures generally offer the best overall performance, balancing high sensitivity (GF > 40) with long-term durability (up to 10,000 cycles) [405, 407]. Self-healing systems exhibit excellent recovery, but suffer from low GF (< 10) and limited stability under high-frequency fatigue [411, 414]. Hierarchical micro-/nanostructures and interpenetrating networks can achieve high GF (> 80) but with lower durability (~ 5000 cycles). However, mechanical reinforcement depends not only on structural design but also on material properties and interfacial bonding [21]. Unfortunately, most research on contact angle stability of superhydrophobic wearable sensors uses static measurements and cannot reflect dynamic wetting robustness. Dynamic stability under cyclic strain depends on micro-/nanostructure design and substrate compatibility; rigid structures easily collapse, and repeated strain causes delamination. Dynamic stability should be a key metric, and future designs require elastic substrates, flexible micro-/nanostructures, and strong interfacial bonding.

Furthermore, despite striking advances under well-defined laboratory conditions, mechanical robustness falters once sensors confront the volatile extremes of real-world duty: Actual load spectra are richer and harsher than any standardized protocol, nucleating failure modes absent in benchtop tests [391]; architectures engineered for maximal stability stiffen the composite, forfeiting the very pliability that defines wearable electronics [291]; and self-healing chemistries demand triggers—solvent, heat, sustained contact—that are seldom available on moving human skin [417, 418]. Bridging this gap will require a rigorously practical suite of mechanical test metrics that systematically recapitulate the stochastic, multiaxial, and long-duration insults encountered in service.

Mechanical assessment protocols must abandon isolated tensile cycles or sandpaper rubs in favor of multifactor, sequentially coupled schemes that replicate authentic wear: (i) multiaxis fatigue combining 20%–30% strain, 10 mm bend curvature, and ± 90° torsion, demanding < 10% resistance drift, < 15% sensitivity shift, and ≤ 5° WCA loss after ≥ 50,000 cycles; (ii) durability in synthetic sweat (ISO 3160-2) cycled 25–45 °C for 10,000 passes with < 20% electrical decay; (iii) impact (50 g, 10 cm drop), scratch (1 kPa Mohs stylus/steel wool), and abrasion (Taber CS-10, 500 g, ≥ 1000 cycles; linear abrader ≥ 50 cycles) while retaining WCA > 150° and < 10% reduction; (ⅳ) for self-healing systems, ≥ 80% mechanical, and ≥ 95% superhydrophobic recovery within 24 h at ambient conditions, sustained over five cuts with fifth-cycle efficiency ≥ 70% of the first. Only devices surviving this regimen can credibly advance from laboratory curiosity to commercial wearable.

### Interfacial Wetting State Transition and Stabilization

The transition of superhydrophobic wearable strain sensors from laboratory demonstrations to engineered applications is impeded by their limited state robustness in real-world environments [346]. Under aggressive conditions such as condensation, freezing, viscous fluids, or oils, Cassie–Baxter state is easily disrupted, leading to critical failure modes: Condensed water or low-surface-tension oils penetrate the micro–nanopores, causing a rapid transition from superhydrophobic to hydrophilic or oleophilic wetting states [321, 419]; and changes in the interfacial physicochemical properties result in markedly stronger adhesion, leading to droplet retention, contaminant accumulation, and even physical damage to the microstructures [37, 322]. This vicious cycle of surface and interfacial degradation directly causes signal drift, sensitivity loss, and ultimately complete functional failure of the sensor. Therefore, developing innovative material designs and structural engineering strategies to confer robust environmental stability has emerged as a key research frontier and an urgent challenge in the field. The following sections systematically review recent strategies and advances in addressing state robustness.

#### Cassie–Wenzel Wetting Transition

Constructing superhydrophobic surfaces with stable microscopic morphologies through advanced fabrication and modification techniques enables precise control over micro-/nanofeatures. These surfaces exhibit pronounced superhydrophobicity, characterized by high contact angles and low sliding angles, which effectively resist wetting by liquids. By mitigating liquid penetration and diffusion into the material, this approach substantially reduces performance degradation and serves as a fundamental strategy for enhancing long-term operational stability under complex environmental conditions [420].

**Fig. 20:**
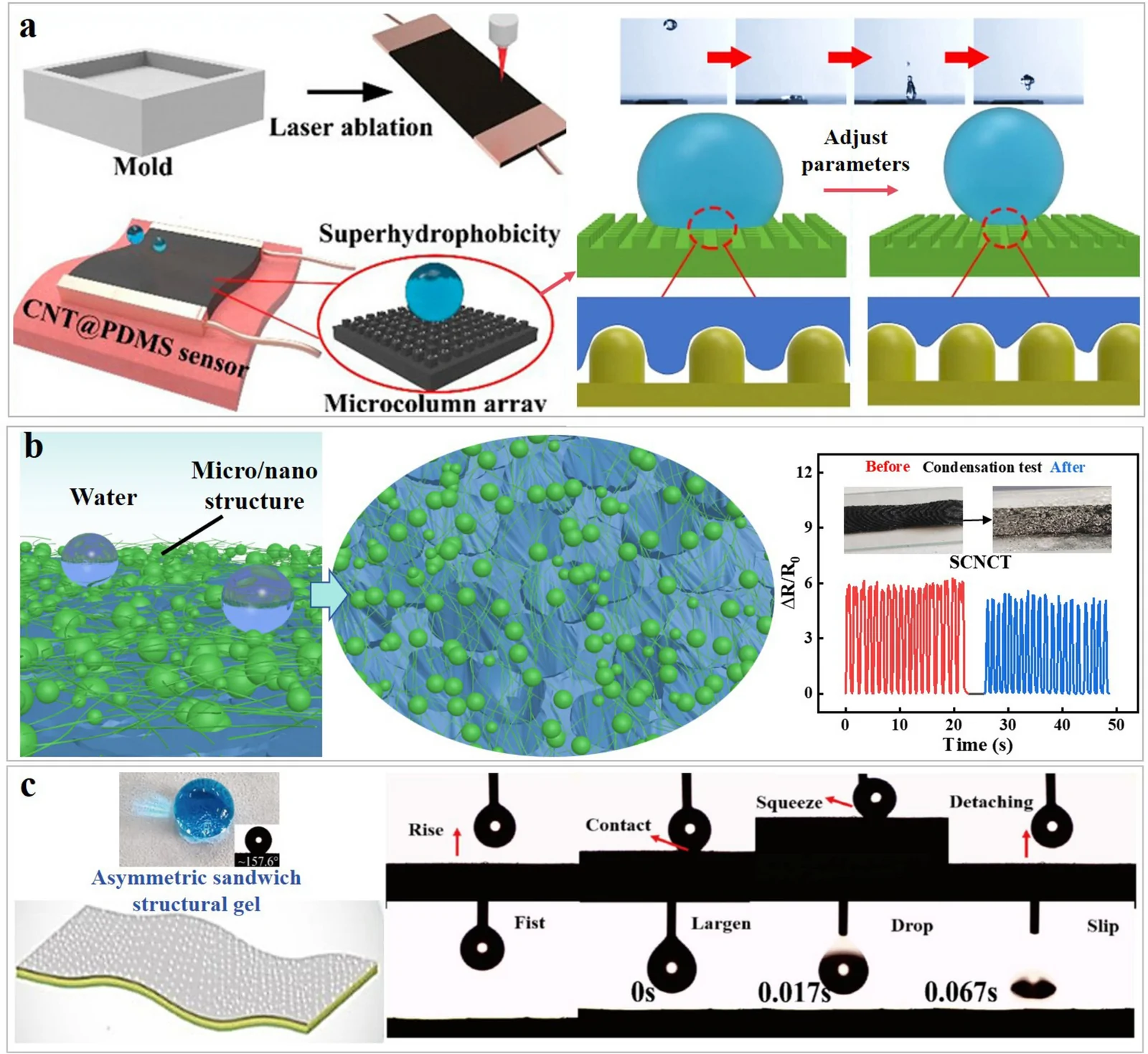
Structural reinforcement. **a** L-CNT@PDMS sensor with picosecond laser-etched micropillar array [421]. Copyright 2024, American Chemical Society. **b** Carbon dot-welded, multidimensional-interlock fabric strain sensor [362]. Copyright 2025, Elsevier. **c** Bioinspired trilayer gel enabling reversible underwater adhesion and antifouling [332]. Copyright 2025, Elsevier

Inspired by biological surfaces such as edelweiss and lotus leaves, researchers have constructed biomimetic micro-/nanocomposite structures. Techniques including direct laser writing and template methods are used to fabricate overhanging and hierarchical rough micro-/nanostructures. For example, the L-CNT@PDMS sensor employs picosecond laser to prepare microcolumn arrays, achieving a superhydrophobic surface with a contact angle > 151° and a sliding angle < 3°, which effectively resists liquid infiltration and exhibits self-cleaning capability (Fig. 20a) [421]. Similarly, the LIG-based biomimetic sensor leverages laser-induced graphene structures on a PDMS/PEEK composite substrate, demonstrating a contact angle of 158° and a sliding angle of 3° while maintaining high sensitivity, with resistance to common liquid contaminants [37]. Additionally, porous interfaces with high roughness and mechanical toughness have been constructed via nonsolvent-induced phase separation or fiber coupling technology. For example, our group designed a carbon dot–line–surface-coupled fabric sensor that forms a multidimensional interlocked structure through the coupling of two-dimensional conductive adhesives with fluorinated carbon nanotubes/microfibers, retaining superhydrophobicity after immersion in strong acids, alkalis, and salt solutions for 3 h, and tolerating extreme conditions such as ultrasonic vibration and condensed water (Fig. 20b) [362]. The AgNPs@sponge composite forms a porous surface via NIPS, integrating the conductive and photothermal properties of silver nanoparticles to achieve dual functions of superhydrophobicity and strain sensing [387]. Asymmetric layered structures can also realize functional separation and synergistic protection. A biomimetic sandwich-structured gel separates a temperature-triggered adhesive layer, an ion-conductive layer, and a superhydrophobic self-cleaning layer, achieving 846.5% stretchability while enabling underwater adhesion switching and antifouling capabilities, suitable for humid and underwater communication scenarios (Fig. 20c) [332]. Current superhydrophobic designs focus on static wettability, but lack structural robustness, long-term stability, and scalable fabrication for reliable wearable applications.

#### Ice Adhesion and Fluid Penetration

Thermal shocks, rapid steam condensation, and the swift nucleation and accretion of ice jointly compromise surface properties and device operation, motivating researchers to craft environmentally responsive superhydrophobic interfaces grounded in a mechanistic grasp of surface chemistry–environment coupling [422, 423]. Through the ingenious design of surface chemical composition and microstructure, such surfaces can acutely sense changes in surrounding environmental parameters [370]. When exposed to adverse environments like condensation or icing, the surface rapidly adjusts its physicochemical properties in response to environmental stimuli, achieving a shift from traditional passive anti-icing to “active defense” [386]. This effectively inhibits the adhesion of condensed water and the nucleation and growth of ice crystals, ensuring the stable performance of material surfaces under complex dynamic environments.

**Fig. 21:**
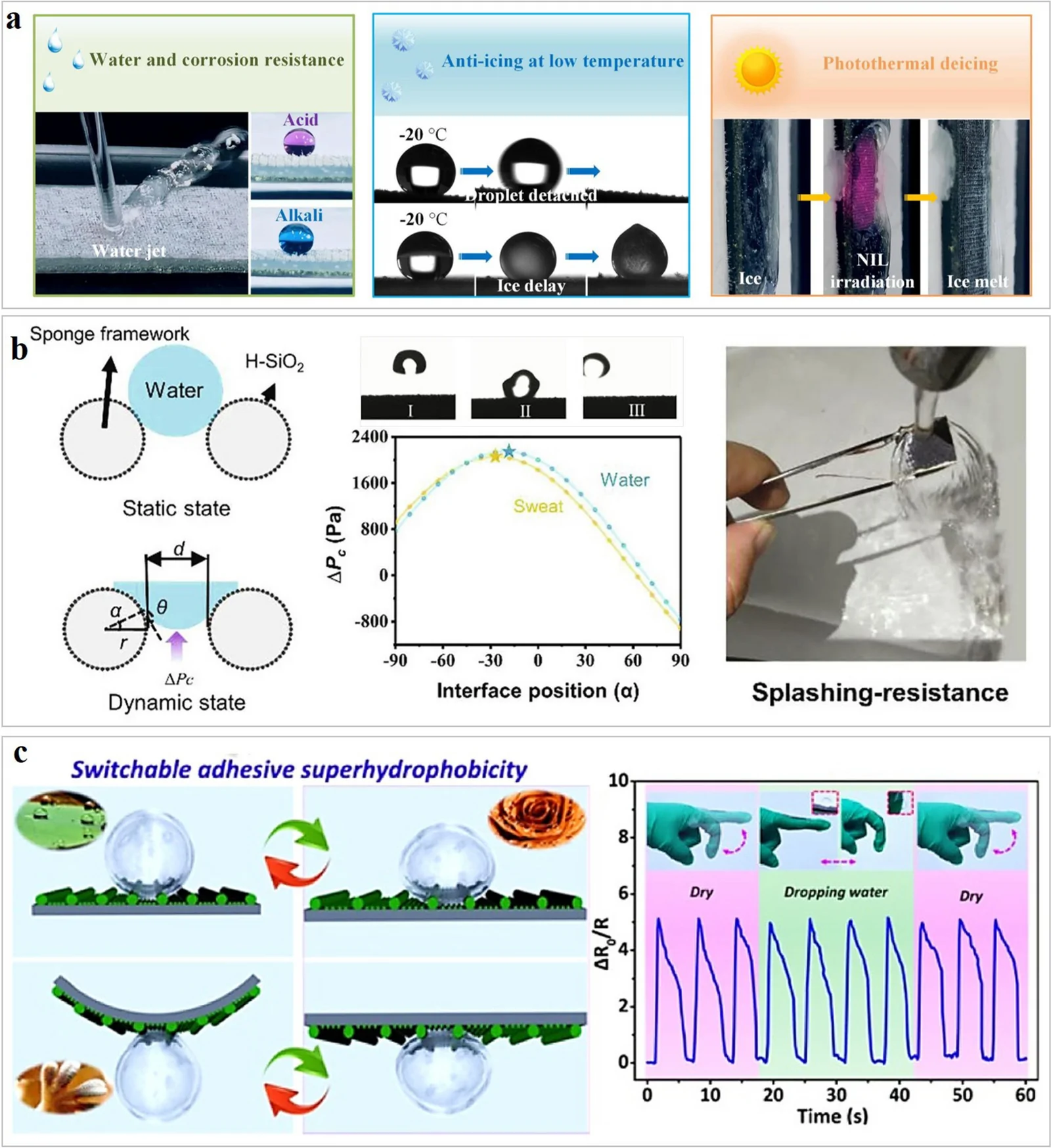
Adaptive surfaces. **a** SR/MWCNTs/LIG/SR sensor with integrated photothermal/Joule heating for on-demand deicing [388]. Copyright 2022, Elsevier. **b** MXene/sodium alginate sponge driven by low-voltage Joule heating to suppress moisture uptake [425]. Copyright 2022, Elsevier. **c** Shape memory superhydrophobic film with switchable adhesion controlled by strain-induced microtexture recovery [429]. Copyright 2021, American Chemical Society

By seamlessly embedding high-efficiency photothermal or high-conductivity electrothermal converters, the sensor surface is driven to evaporate or melt and roll off liquid contaminants in real time [352, 356]. Localized and precise heating is achieved through the Joule effect or photothermal conversion effect. By interrupting the thermodynamic cycle of vapor condensation and subsequent freezing, this protocol retards heterogeneous ice nucleation and accelerates melt water removal, establishing a photothermal/electrothermal platform that affords synergistic anti-icing and antifog performance under sub-zero conditions [424]. This strategy boasts advantages including fast response speed, strong temperature controllability, and compatibility with flexible substrates, providing core support for the stable operation of sensors under extremely low-temperature and high-humidity conditions. For example, the SR/MWCNTs/LIG/SR multilayer composite sensor enhances energy conversion efficiency through a dual conductive photothermal network constructed by MWCNTs and LIG (Fig. 21a) [388]. At a low temperature of – 5 °C, its surface icing time is extended to 36 min compared with traditional superhydrophobic sensors, and rapid deicing within 88 s is achieved under NIR irradiation, while maintaining excellent mechanical flexibility and sensing robustness. Another type of MXene/sodium alginate sponge sensor utilizes the high electrical conductivity of MXene and the porous structure of sodium alginate sponge (Fig. 21b). Rapid local temperature elevation is realized via low-voltage Joule heating requiring only a few volts, effectively inhibiting the adsorption and condensation of water molecules on the sensor surface in high-humidity environments, and ensuring stable resistance response and superhydrophobicity even in saturated water vapor [425].

Leveraging the stimulus-triggered deformation of shape memory polymers or the conformational tunability of responsive molecules, dynamically switchable surface chemistries have been engineered that toggle wettability on demand, yielding smart superhydrophobic interfaces whose wetting state can be reversibly programmed in situ [426]. The core of this strategy lies in triggering the reconstruction of material microstructures or the adjustment of molecular arrangements through mild external stimuli, thereby dynamically regulating surface roughness and interfacial energy to achieve flexible adaptation between superhydrophobic states and low-adhesion properties, and thus adapting to dynamic fluctuations in environmental humidity [427, 428]. For example, thermally or mechanically driven shape memory superhydrophobic films can reversibly adjust the arrangement density and roughness of surface micro-/nanostructures during stretching–recovery cycles via pre-programmed shape memory effects (Fig. 21c). The engineered surface exhibits switchable, adhesion-tunable superhydrophobicity coupled with autonomous “dynamic dehumidification.” Structural actuation displaces residual droplets under elevated humidity or rainfall, suppresses interfacial stiction, and prevents liquid-induced signal drift, thereby guaranteeing sensor reliability across fluctuating moisture environments [429]. Most existing responsive superhydrophobic interfaces rely on photothermal/electrothermal or shape‑memory effects, but lack long‑term durability under repeated thermal/mechanical cycling and fail to fully address structural degradation and signal drift in extreme humid and freezing environments.

#### Oils and Biofouling Resistance

In practical applications, surface contamination caused by oil fouling and bioadhesion is prevalent across various fields [397]. The surfaces of medical devices are prone to adhesion by biomolecules such as blood and proteins, raising the risk of infections and impairing device performance. Surface contamination undermines material performance, incurs substantial economic losses, and introduces acute safety liabilities [419]. Therefore, constructing self-cleaning surfaces has emerged as a key measure to maintain the long-term robustness of materials, addressing surface contamination induced by oil fouling, bioadhesion, and related factors.

**Fig. 22:**
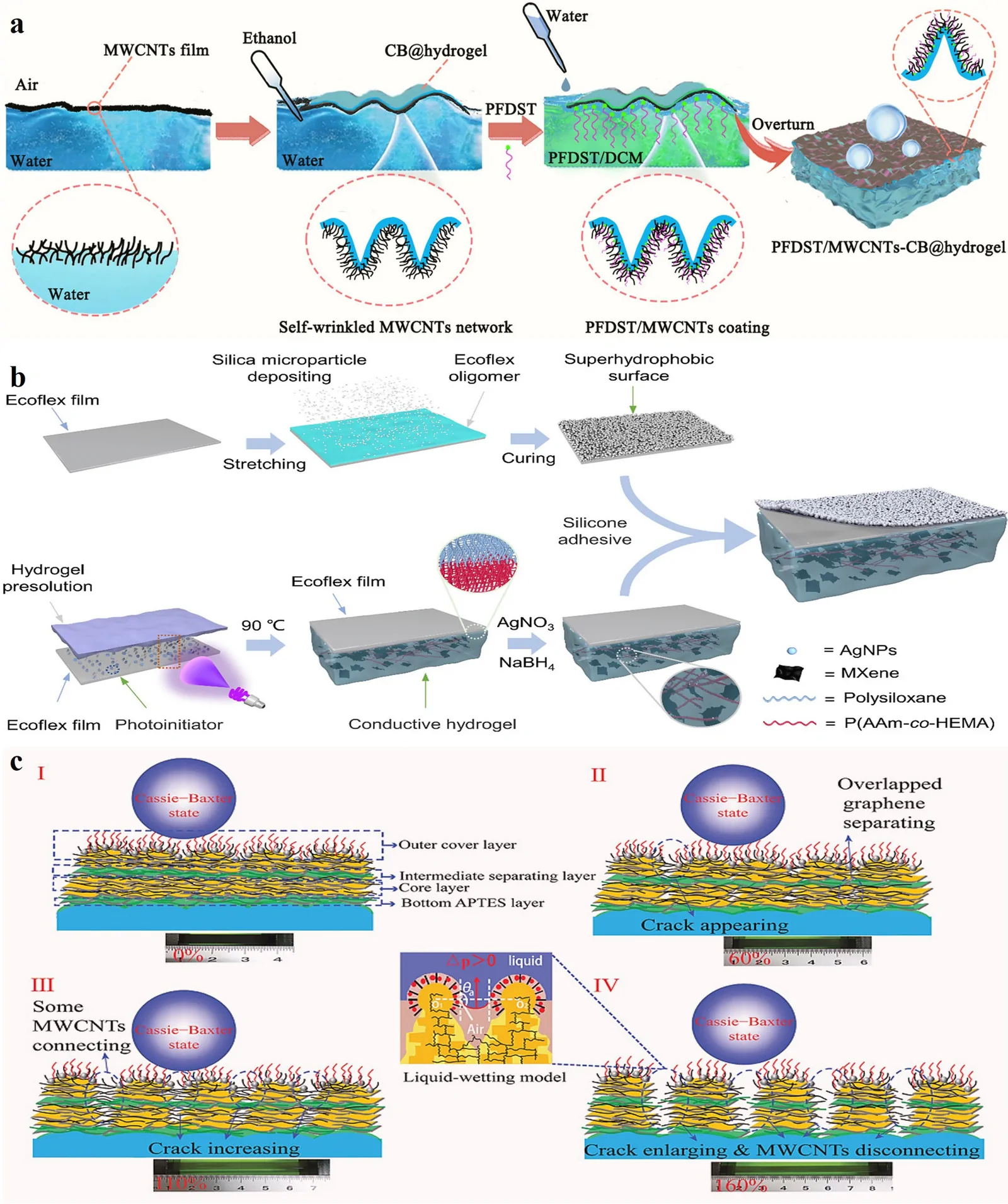
Antifouling surfaces. **a** PFDST/MWCNTs–carbon black@hydrogel sensor showing superamphiphobicity to both water and oil [330]. Copyright 2025, Elsevier. **b** Ecoflex/SiO_2_–hydrogel sensor integrating antibacterial agents with a superhydrophobic texture [347]. Copyright 2021, American Chemical Society. **c** FAMG sensor exhibiting underwater superoleophobic and antibacterial dual functions [346]. Copyright 2020, John Wiley and Sons

Reliable sensing in hostile milieus is secured through a multifunctional guard that unites micro-/nanotopography for physical repulsion with ultralow surface energy and intrinsic chemical inertness [430]. Through their synergistic effect, a superamphiphobic surface with strong repellency to both water and oil is constructed—ultralow surface energy weakens the intermolecular forces between liquids and the interface, while micro-/nanostructures further enhance physical barrier and trap air films, collectively inhibiting the wetting and spreading of both aqueous and oil phases [431]. For example, the sensor based on PFDST (perfluorodecyltriethoxysilane)-modified MWCNTs–carbon black composite hydrogel relies on the synergistic interface constructed by fluorination modification and conductive fillers [330]. Even under a large tensile strain of 70%, its contact angle for water and oil remains at 160°, demonstrating omnidirectional and durable antiwetting capability. This effectively avoids signal drift caused by the intrusion of oil-based media into the conductive network, enabling the sensor to stably adapt to complex application scenarios where mechanical deformation and oil-containing environments coexist (Fig. 22a).

On the other hand, a synergistic “superhydrophobic physical shield plus antimicrobial chemical inhibition” strategy embeds biocidal moieties directly into the micro-/nanotopography, merging passive repellency with active pathogen suppression [311]. Superhydrophobic structures inhibit initial adhesion by reducing the contact area between microorganisms and the interface, while antibacterial components disrupt microbial cell membranes or metabolic processes by releasing active substances, achieving efficient antibioadhesion through the dual effects [432]. In the Ecoflex/SiO_2_–hydrogel sensor, AgNPs preserve the superhydrophobic microtexture while releasing Ag⁺ ions that potently inhibit Staphylococcus aureus and Escherichia coli proliferation, suppressing biofilm formation and extending operational lifetime in microbially contaminated environments (Fig. 22b) [347].

Similarly, F/Ag/MWCNT/G-PDMS (FAMG) sensor achieves a synergistic effect through the antibacterial function of silver nanoparticles and the low-surface-energy modification of fluorosilane, simultaneously achieving dual core functions of underwater superoleophobicity and antibacterial activity (Fig. 22c). Moreover, it maintains stable mechanical flexibility and sensing response performance under various dynamically challenging environments such as simulated rainfall erosion and bacterium-containing droplet impact, highlighting the adaptability of this synergistic strategy to complex multimedium environments [346]. However, current studies cannot achieve long-term stability under complex composite pollution such as the coexistence of oil, proteins, and microorganisms, nor can they realize durable self-cleaning and sensing stability under high-strength mechanical deformation.

To address state instability caused by condensation, icing, oil contamination, and biofouling, research on superhydrophobic wearable strain sensors has converged along three distinct reinforcement pathways: first, structural reinforcement, which employs biomimetic micro–nanostructures to form physical barriers that passively resist liquid penetration, establishing a robust foundation for state robustness [421]; second, functional surfaces, which incorporate photothermal/electrothermal elements or shape memory materials to enable active deicing, anticondensation, and dynamic moisture removal [425]; and third, antiadhesion surfaces, which utilize fluorination to achieve omniphobicity or integrate antibacterial components, directly countering the sources of oil and biological contamination to confer self-cleaning capability [347].

Yet these tactics falter once confronted with the open-air continuum: Real-world effluents arrive as relentless chemical cocktails that far exceed single-factor laboratory vignettes, irrevocably clogging or poisoning micro-/nanotextures [31]; active countermeasures demand more energy and reliability than a wearable battery can spare, rendering sustained photothermal or electrothermal deicing both impractical and prone to cyclic degradation [433]; and the same ultralow-surface-energy chemistry that repels contaminants inherently sacrifices wear resistance, coupling antifouling prowess to mechanical fragility [33].

To advance practical adoption, rigorous and realistic evaluation standards for state robustness must be established, including dynamic pollution durability tests where sensors maintain WCA decrease < 10% and sliding angle attenuation < 50% after ≥ 100 cycles of alternating simulated sweat and dust pollution, low-power deicing efficiency standards requiring autonomous deicing and signal resumption within 120 s in – 10 °C and 90% humidity conditions, and antibiological contamination tests reducing bacterial adhesion ≥ 99% with sensing signal drift < 5% after 7 d immersion in protein- and bacteria-rich simulated body fluids, as only such quantitative standards simulating real service conditions can effectively screen devices with high environmental robustness and accelerate their transition from laboratory to practical application.

To systematically summarize the failure mechanisms and corresponding mitigation strategies, Table 3 classifies the key bottlenecks into chemical stability, mechanical robustness, and wetting state, and outlines the state-of-the-art solutions. This table provides a concise overview for readers to understand and apply the design principles of robust amphibious flexible sensing systems.

**Table 3:** Failure mechanism and mitigation strategy

Section	Failure type	Intrinsic instability mechanism	Mitigation strategy	References
3.1 Chemical destabilization	Corrosive Medium Attack	1. Coatings hydrolyze and oxidize; 2. Networks oxidize and fracture; 3. Corrosion removes air cushion	1. Construct hierarchical conductive structures; 2. Modify PDMS to protect networks; 3. Anchor nanostructures via PDA	[297, 362, 367, 369]
	UV Irradiation Degradation	UV breaks Si–O bonds; Polymers photo-oxidatively degrade; Functional loss causes hydrophilicity	1. Use photo-oxidation-stable matrices; 2. Incorporate UV shielding components; 3. Design coating-free superhydrophobic structures	[145, 371–373]
	Extreme temperature failure	High temperature: 1. Mismatch induces interfacial cracking; 2. Coatings thermally decompose Low temperature: 1. Network embrittlement causes coating failure; 2. Crystallization ruins microstructures	High temperature: 1. Heat-resistant frameworks; 2. Add flame-retardant inorganic components Low temperature: 1. Antifreeze hydrogels; 2. Thermal management for deicing; 3. Bilayer structure	[377–389]
3.2 Mechanical Failure	Dynamic Deformation Fatigue	1. Cyclic stretching causes crack propagation; 2. Interfacial debonding disrupts pathways; 3. Fatigue causes superhydrophobicity loss	1. Covalent cross-linking interfacial bonding; 2. PDA enhances layer–substrate adhesion; 3. Fatigue-resistant gradient wrinkle structure design; 4. Elastic microstructures for stress buffering	[376, 402, 405, 406]
	Wear and peel resistance	1. Friction/bending destroys structures; 2. Delamination causes dual performance loss	1. Multilevel wrinkle/buckling structures; 2. Core–shell/multilayer structures; 3. Synergistic dual conductive networks for reinforcement;	[21, 299, 334, 407]
	Insufficient Self-Healing Capacity	1. Properties unrecoverable post-damage; 2. Asynchronous recovery dual properties	1. Self-healing matrices with dynamic reversible bonds; 2. Hydrophobic segments reconstruct surface energy; 3. Synchronous recovery of conductive and superhydrophobic properties	[411, 412, 414, 415]
3.3 Interfacial Wetting State Transition	Cassie–Wenzel transition	1. Pressure/condensation drives liquid infiltration into microstructures; 2. Irreversible wetting transition causes sensor signal drift	1. Biomimetic overhanging/hierarchical micro-/nanostructures for air retention; 2. Porous interlocked structures resist liquid penetration; 3. Asymmetric layered structures for functional separation	[37, 332, 362, 421]
	Ice Adhesion and Fluid Penetration	1. Condensation/icing destroys the air cushion layer; 2. High-pressure fluid penetrates microstructures	1. In situ deicing/antifogging via photothermal/electrothermal effects; 2. Dynamic wettability tuning using shape memory polymers; 3. Active thermal control to suppress water molecule adsorption	[388, 425, 429]
	Oil and Biofouling Resistance	1. Oil/protein/bacteria adhesion clogs microstructures; 2. Interfacial contamination results in signal distortion	1. Omniphobic fluorination modification; 2. Superhydrophobic + Ag^+^ synergistic sterilization 3. Photocatalytic self-cleaning for pollutant degradation	[318, 346–348]

### Multi-Instability Coupling and Evaluation Framework

#### Multi-Instability Coupling Mechanisms

The failure of superhydrophobic wearable strain sensors arises from the synergistic coupling of chemical instability, mechanical damage, and wetting state transition rather than any single factor. This core mechanism critically limits sensor service life and requires systematic elucidation. Their coupling exhibits a chain-triggering and vicious cycle pattern. This subsection elucidates these coupling mechanisms and highlights a critical gap in the literature: Most existing studies focus on isolated failure factors [31].

Mechanical damage, such as cyclic stretching or coating delamination, typically serves as the initial trigger. Mechanical deformation disrupts the superhydrophobic micro-/nanostructure, causing loss of the trapped air layer and inducing wetting state failure via an irreversible Cassie–Wenzel transition [434, 435]. Simultaneously, exposed conductive networks and substrates become more susceptible to chemical media like sweat electrolytes, accelerating oxidation of conductive materials and hydrolysis of polymer chains [436]. Compared with static immersion in corrosive media alone, this coupling effect leads to substantially faster performance degradation. Notably, most existing studies evaluate chemical stability only under static conditions or mechanical durability only under dry conditions, overlooking this critical coupling effect.

Conversely, chemical instability degrades interfacial adhesion and mechanical strength, increasing susceptibility to mechanical damage under strain. Wetting state failure further weakens interlayer bonding and exacerbates the coupling of chemical degradation and mechanical fatigue [437]. Biological stresses such as bacterial adhesion and biofilm formation alter surface wettability and mechanical properties of sensors, thereby exacerbating mechanical failure. Bacterial colonization and biofilm growth weaken structural integrity [438]. Under cyclic stretching, biofilm-covered regions suffer severe coating delamination and crack propagation, degrading mechanical durability. However, few studies integrate biofilm evolution with mechanical fatigue testing, leading to over-estimated robustness in real wearable scenarios.

Therefore, developing robust superhydrophobic wearable strain sensors requires moving beyond single-mode optimization toward a multidimensional synergistic strategy. Disrupting the coupled failure chain significantly enhances long-term sensor reliability.

#### Standardized Evaluation Parameters

Although existing literature has established evaluation metrics for chemical stability, mechanical durability, and wetting state stability, a unified testing protocol remains lacking. This is primarily due to three factors: the wide variation in application scenarios—from health care to outdoor sports to underwater operations—each with distinct robustness requirements [439, 440]; inconsistencies in testing setups and methods; and the predominance of isolated research efforts without coordinated involvement from industry, institutions, and enterprises, leading to the use of self-defined parameters. Key testing parameters such as UV exposure time, cyclic strain cycles, strain amplitude, corrosive medium concentration, washing conditions, and extreme temperature ranges vary considerably across representative studies, which hinders fair cross-study comparison and reliable benchmarking, may lead to performance over-estimation, and creates a disconnect between laboratory testing and practical application.

To address this lack of consensus, this article proposes standardized testing parameters and evaluation requirements based on industry standards, scenario-specific demands, and testing equipment practicality. The proposed standards for chemical, mechanical, and interfacial wetting states are established on three foundations: reference to existing industry standards, statistical analysis of published literature, and practical engineering objectives of wearable sensors, thereby ensuring scientific rigor and operability.

##### Chemical robustness

For the UV irradiation test, we refer to the ISO 4892-3 standard for plastic UV aging, where a 100 h exposure is equivalent to approximately one year of outdoor exposure based on an average daily UV exposure of six hours; the test parameters include a wavelength of 365 nm (UVA, simulating outdoor natural light), an irradiance intensity of 10 mW cm^−2^, and an irradiation duration of 100 h, with evaluation indicators being a contact angle decrease of less than 10%, a resistance drift of less than 15%, and no obvious cracking [441]. For the corrosive medium immersion test, 3.5 wt% salt water is used to simulate seawater salinity, while strong acid and strong alkali solutions are represented by HCl at pH 1 and NaOH at pH 14, respectively [442, 443]; simulated sweat follows the ISO 3160-2 and ISO 10993-15 standards (Na^+^ 100 mmol L^−1^, K^+^ 20 mmol L^−1^, Cl^–^ 110 mmol L^−1^), covering scenarios such as outdoor use, medical applications, and underwater environments, with evaluation indicators requiring a contact angle of ≥ 150° and a resistance drift of < 10% after 24 h of immersion, referring to ASTM F1868-07. For the extreme temperature cycling test, referring to the ASTM D638 standard, the test parameters include a low temperature of –20 °C, a high temperature of 120 °C, a holding time of 2 h at each temperature point, and 50 cycles to cover most outdoor and industrial environments, with the evaluation standard being an elongation at break retention of ≥ 80% after cycling and no failure in hydrophobic performance.

##### Mechanical Robustness

In the cyclic strain test, referring to the actual use scenarios of wearable devices such as joint bending and limb movement, the test parameters include a strain amplitude of 20% representing the common deformation range for daily wear, a frequency of 1 Hz, and 10,000 cycles according to ISO 13934-1, with evaluation indicators requiring a resistance drift of less than 10%, no conductive network fracture or coating peeling, and a contact angle of at least 150° [274]. In the wear test, a Taber abrasion tester with a CS-10 abrasive wheel is used under a load of 500 g for 1000 wear cycles per ASTM D4060, while linear friction with #400 sandpaper is performed under a pressure of 1 kPa for 50 cycles to simulate clothing friction and skin contact in daily wear; after wear, the contact angle must remain at least 150° and the resistance drift below 20%. In the peel test, 3 M tape is applied at a pressure of 1 kPa and then peeled off at 180° according to ASTM D3359 for 1000 peel cycles, with evaluation standards being a resistance drift of less than 20% and a contact angle of at least 150°. In the bending and torsion test, which simulates the extreme deformation of joint movements such as wrists and fingers, the requirements are a bending radius of 10 mm based on human joint dimensions, a bending angle of ± 90° for 5,000 cycles, and a torsion angle of ± 90° for 5,000 cycles; after the test, there should be no structural damage, with a resistance drift below 10% and a contact angle of at least 150°.

##### State Robustness

The Cassie–Wenzel transition test requires a liquid pressure of 0.1 MPa to simulate raindrop impact and shallow underwater immersion, corresponding to the pressure at a water depth of 10 m, along with a low-surface-tension liquid composed of 30% ethanol by volume to simulate complex liquid environments such as oil stains and sweat, with the evaluation standard being no irreversible wetting transition and a contact angle of at least 145°. In the anti-icing and anticondensation test, the parameters are set at a temperature of – 10 °C and a humidity of 90% to cover cold and high-humidity environments, with a condensation time of 2 h; the ice adhesion test load is 1 N referring to the ASTM C1654 standard, and the evaluation standard requires no obvious icing and an ice adhesion strength of less than 0.2 N cm^−2^. In the antibacterial test, “Escherichia coli” and “Staphylococcus aureus” with a concentration of 10^6^ CFU mL^−1^ are used, and the immersion time is 72 h according to the ISO 22196 standard for plastic antibacterial testing, with the evaluation standard being an antibacterial rate of at least 99% and no biofilm formation [444].

## Conclusions and Outlook

Superhydrophobic wearable strain sensors have emerged as a pivotal category of wearable electronics, with the core goal of reconciling high sensing performance—encompassing sensitivity, stretchability, and response speed—with robust environmental adaptability. This review systematically summarizes recent advances in this field, covering design principles, material innovations, structural engineering, and functional integration. The synergy between flexible substrates and conductive components lays the foundation for flexible conductive sensors [44]. Carbon-based materials like graphene and MXene stand out due to their robustness, flexibility, and piezoresistive properties [135], while conductive polymers [204] and metallic nanomaterials [130] offer high conductivity and processability. Structural designs—such as micro-/nanohierarchical rough surfaces and biomimetic architectures [421]—combined with self-healing systems enhance superhydrophobicity, mechanical strength, and durability [418]. Functional integration further enables antifouling [322], underwater monitoring [335], and antibacterial performance [345], allowing reliable operation in complex environments such as sweat, oil, and biofluids.

Notably, substantial progress has been made in addressing three core stability challenges: Chemical robustness is enhanced via corrosion-resistant coatings as well as UV/thermal shielding components [362, 370]; mechanical robustness is improved through interfacial modification like PDA, structural reinforcement including wrinkled and buckled structures, and self-healing mechanisms [398, 402, 405, 415]; state robustness is achieved by structural optimization such as porous and interlocked networks, environmentally responsive surfaces including photothermal and electrothermal deicing, and antiadhesion designs like superamphiphobicity and antibacterial components [347, 387, 424]. Despite these advances, critical bottlenecks remain: insufficient long-term stability under multifactor coupled harsh environments such as cyclic mechanical stress combined with chemical corrosion and biological contamination, lack of standardized evaluation systems for multidimensional robustness covering chemical, mechanical, and state aspects, and practical hurdles in low-cost scalable fabrication of high-uniformity sensors.

To address these bottlenecks, future research must pivot toward a holistic strategy for environmental resilience. This demands a fundamental shift in material design from single-function solutions to multi-mechanism architectures that synergistically integrate self-healing chemistry, mechanically durable frameworks, and stable surface repellency. Building upon this material foundation, the field must establish standardized, multifactor evaluation protocols that accurately replicate the coupled chemical–mechanical–biological stresses of real-world scenarios. Furthermore, the transition from laboratory concept to reliable technology hinges on developing scalable manufacturing processes capable of faithfully replicating these critical micro–nanotextures at commercial scale. Ultimately, the success of this integrated approach must be validated through demonstration in demanding application scenarios, such as prolonged underwater monitoring, harsh weather tracking, and durable human–machine interfaces for extreme environments.
